# Factors associated with handgrip strength across the life course: A systematic review

**DOI:** 10.1002/jcsm.13586

**Published:** 2024-08-26

**Authors:** Leticia W. Ribeiro, Sara Berndt, Gregore I. Mielke, Jenny Doust, Gita D. Mishra

**Affiliations:** ^1^ School of Public Health The University of Queensland Brisbane Queensland Australia

**Keywords:** Muscle strength, Life course perspective, Ageing, Risk factors, Muscle weakness

## Abstract

**Background:**

Muscle strength is essential for healthy ageing. Handgrip strength (HGS) has been recommended by expert bodies as the preferred measure of muscle strength, in addition to being considered a strong predictor of overall health. Cross‐sectional studies have shown several potential factors associated with HGS, but a systematic review of factors predicting HGS over time has not previously been conducted. The aim of this study is to systematically review the literature on the factors associated with adult HGS [at follow‐up(s) or its rate of change] across the life course.

**Methods:**

Searches were performed in MEDLINE via Ebsco, Embase and SPORTDiscus databases. Longitudinal studies assessing potential factors impacting adult HGS over time were included in the analyses. Based on previously established definitions of consistency of results, a semiquantitative analysis was conducted using the proportions of studies supporting correlations with HGS.

**Results:**

A total of 117 articles were included in this review. Factors associated with HGS were grouped into 11 domains: demographic, socioeconomic, genetic, early life, body composition, health markers/biomarkers, health conditions, psychosocial, lifestyle, reproductive and environmental determinants. Overall, 103 factors were identified, of which 10 showed consistent associations with HGS over time (i.e., in at least four studies with ≥60% agreement in the direction of association). Factors associated with greater declines in HGS included increasing age, male sex, higher levels of inflammatory markers and the presence of cardiovascular diseases. Education level, medication use, and self‐rated health were not associated with the rate of change in HGS. Increased birth weight was associated with a stronger HGS over time, whereas depressive symptoms were linked to a weaker HGS, and smoking habits showed null associations.

**Conclusions:**

Comparison between studies and estimation of effect sizes were limited due to the heterogeneity in methods. Although sex and age may be the main drivers of HGS decline, it is crucial to prioritize modifiable factors such as inflammation and cardiovascular diseases in health interventions to prevent greater losses. Interventions to improve birth weight and mental health are also likely to produce positive effects on muscle strength. Our results point to the complexity of processes involving muscle strength and suggest that the need to better understand the determinants of HGS remains.

## Introduction

The World Health Organization describes healthy ageing as ‘the process of developing and maintaining the functional ability that enables wellbeing in older age’.^1^ Muscle strength is important for functional independence, and a predictor of healthy ageing.^2^, ^3^ Handgrip strength (HGS) is a frequently used measure of muscle strength, endorsed by expert bodies in sarcopenia and frailty, as it is a non‐invasive procedure, it is relatively low cost and correlates well with other physical functioning measurements.^3^, ^4^, ^5^ Low HGS has been associated with an increased risk for a range of adverse health outcomes with ageing, including sarcopenia, frailty, cardiovascular diseases, type 2 diabetes, metabolic syndrome, cognitive decline and all‐cause mortality.^2^, ^6^

HGS peaks at around the age of 30 years, remains stable until about the ages of 40 to 50 years and starts to decline thereafter.^7^, ^8^ Nonetheless, advancing age alone cannot explain the decline in muscle strength after the fifth decade of life.^9^ There have been multiple factors shown to be associated with HGS in cross‐sectional studies; however, findings are often mixed.^3^, ^10^ Moreover, the predictors of HGS over time remain largely unknown. To the best of our knowledge, there is no comprehensive review of the factors associated with HGS in adulthood.

Muscle strength and physical function in older life reflect not only the age‐related rate of decline rather the peak reached during the earlier stages of life.^11^, ^12^ A life course approach to physical functioning may provide a better understanding of the trajectories of functional ability and enable planning prevention strategies during people's lifetime.^11^ Therefore, this review aims to understand the factors that determine adult muscle strength, measured by HGS, across the life course. We analysed all possible HGS outcomes, including measures of HGS in young to late adulthood, and the rate of decline in HGS over time.

## Methods

This review is reported in line with the Preferred Reporting Items for Systematic‐Reviews and Meta‐Analyses (PRISMA). A protocol for the review was registered in the International Prospective Register of Systematic Reviews (PROSPERO ID: CRD42022374356).

### Search strategy

We included MeSH or other subject terms and synonyms in the search string. The search was designed with the help of a librarian. The following automation tools were used in the design of the search: Polyglot Search Translator^13^ and SearchRefinery.^14^

Searches were run in the following databases: MEDLINE via Ebsco, Embase and SPORTDiscus. Searches were run from inception to 4th October 2022 (see Appendix S1A for the search strategies used in each database).

Restrictions were applied to the publication types. Conference abstracts, books, book chapters and thesis were purposefully removed from the search results. No language or publication date restrictions were applied to the search. However, reports in languages other than English, Japanese, Spanish or Portuguese were excluded.

### Study selection and screening

We identified and included prospective studies that assessed factors associated with HGS in adulthood. Studies were included if the following criteria were met:

- Epidemiological studies of non‐clinical populations conducted in the general community (e.g., people's homes, community facilities, sheltered housing complexes and retirement villages).
- Assessed the prospective association of one or more exposure variables with HGS during adulthood (18+ years old).
- Described either HGS at follow‐up(s) or the rate of change in HGS over the follow‐up period as the main outcome.

Studies that analysed grip strength as the exposure variable (e.g., that examined the association between changes in grip strength associated with reduced morbidity and mortality) or that were compared with any intervention (e.g., changes in grip strength after exercise training or dietary supplementation) were not included. The initial search returned 5044 studies and 155 were selected. Studies with sample sizes smaller than 560 (i.e., <25th percentile) were excluded because smaller samples can be less representative and yield less meaningful results.

Two authors (L. R. and S. B.) independently screened the titles and abstracts against the inclusion criteria. One review author (L. R.) retrieved the full text, and two authors (L. R. and S. B.) screened the full text for inclusion. Discrepancies were resolved by consensus or by referring to a third author. We used these automation tools to help with the screening of articles: Screenatron/Disputatron.^15^

### Data extraction

Data were extracted by two authors (L. R. and S. B.) using a standardized form based on the Critical Appraisal and Data Extraction for Systematic Review of Prediction Modelling Studies (CHARMS) checklist.^16^ The following data for study characteristics were extracted from each included study: study design, participants (e.g., eligibility and recruitment method; age and gender distribution; duration of follow‐up), sample size, outcome (e.g., definition and method for HGS measurements), factors included in the analyses and statistical methods used (e.g., type of model, model evaluation and handling of missing data) and results.

### Quality and risk of bias assessment

The Quality in Prognosis Studies (QUIPS) tool^17^ was used for a systematic appraisal of bias in the studies. The justification for using the QUIPS tool lies in its detailed assessment of potential prognostic (or exposure) factors associated with an outcome of interest over time, of the outcome measurement itself, confounding factors and model analysis, offering a more comprehensive evaluation compared with other available tools. Two reviewers were involved in the quality assessment (L. R. and S. B.). Discrepancies were discussed between the two reviewers and/or solved by a third reviewer. Each study was assigned an overall risk of bias (high, moderate or low) based on the rating of the report of six areas, including study participation, study attrition, prognostic factor measurement, outcome measurement, study confounding and statistical analysis and reporting.

### Analysis of studies

We grouped potential factors into 11 domains: demographic, socioeconomic, genetic, early life, body composition, health markers/biomarkers, health conditions, psychosocial, lifestyle, reproductive and environmental determinants. Then, tables were created to provide a summary of the state of the literature for each factor associated with HGS, which allows for checking the consistency of the associations between studies. Conceptually similar factors were grouped, even if they were not measured in the same way. We separated variables with different exposure times, such as weight (single point in time) and weight history (multiple points in time).

Based on previous reviews in physical activity and HGS,^18^, ^19^, ^20^ an estimate was calculated of the proportion of studies indicating a correlation relative to the overall number of studies found within each variable. When 60–100% of studies supported a positive, negative or no association at all the symbols ‘+’, ‘‐’ and ‘0’ were used, respectively. Additionally, when there were four or more studies indicating such associations, it was coded as ‘++’, ‘−−’, or ‘00’, to indicate that the variable has been frequently studied and that there was consistency in the results. When there was a lack of consistency in the findings (i.e., 0–59% studies supporting the correlation), non‐linear associations or mainly sex‐specific associations the code ‘mixed’ was used. If the results were male or female‐specific, ‘M’ or ‘F’ was indicated next to the article's reference number. ‘I’ and ‘II’ were used to indicate studies that had an entire sample of males and females, respectively.

Given the substantial heterogeneity, a meta‐analysis was inappropriate as it would not provide meaningful or reliable results. The studies varied in key aspects such as the protocol for measuring HGS, the age and sex distribution of the sample and the covariates considered. Differences in HGS protocol include the type of measurement (average, maximal, summed or relative HGS), hands measured (both, dominant, non‐dominant and preferred), the test trials^1^, ^2^, ^3^, ^4^, ^5^, ^6^, ^7^, ^8^ and the unit of measurement (kg, N, psi, bar and kPa). Moreover, a variety of statistical methods was used in data analyses, such as correlation tests, analysis of variance (ANOVA) and covariance (ANCOVA), latent growth models, and most commonly, regression analysis, including linear, linear mixed‐effects, logistic, generalized estimating equation and hierarchical models. In addition, cut points for weakness and declines in HGS were frequently not described and derived from data of the studied samples and thus are not generalizable to other populations. Although we may show a combination of results across studies in terms of annualized change in HGS, for example, it is important to note that those results should be interpreted with caution.

## Results

**Figure 1:**
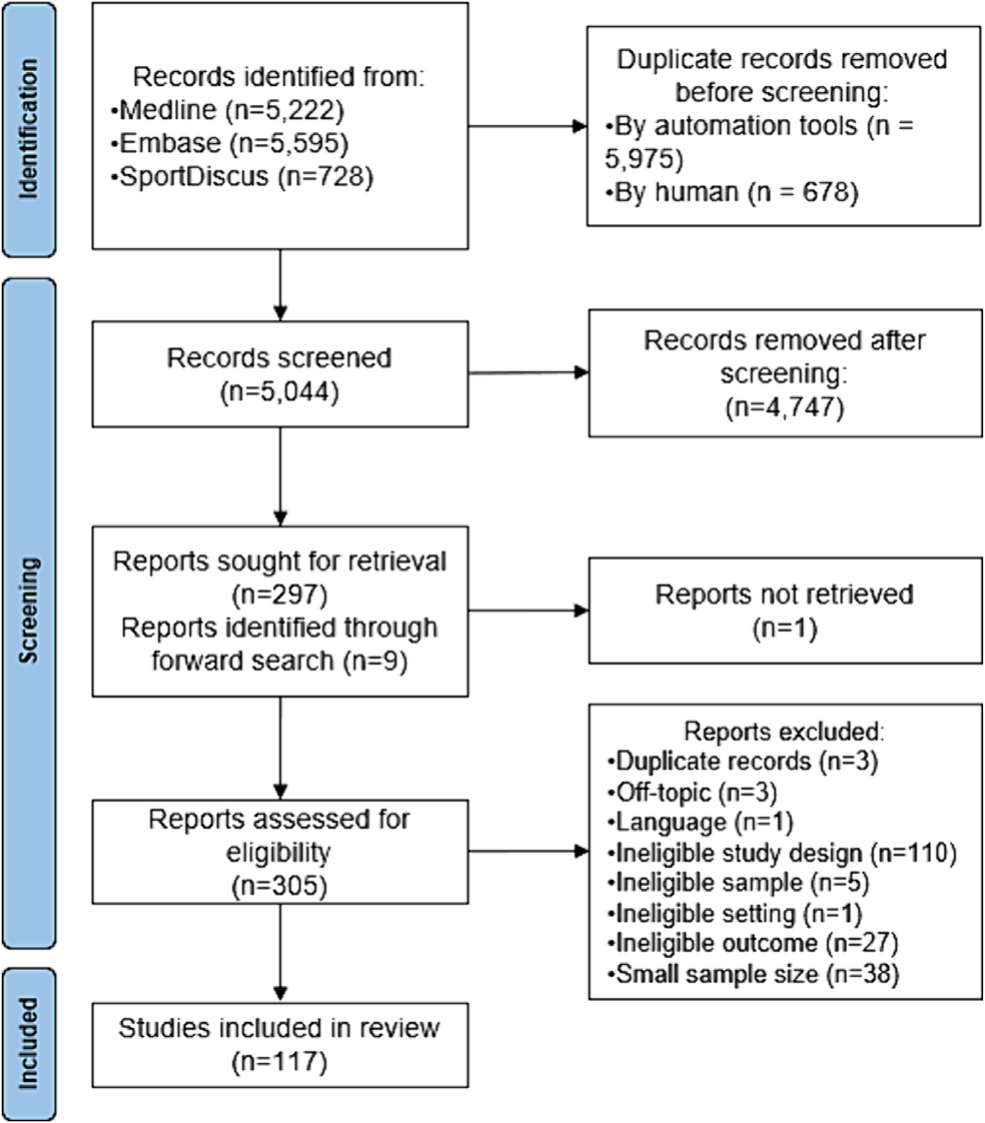
Flowchart of the study selection procedure.

A total of 117 longitudinal studies were included in this review, with a median follow‐up time of 7 years (IQR: 4–12) (*Figure* *1*). Fifty per cent of studies (*n* = 59) reported the associations between the exposure variables and the rate of change or the decline in HGS (e.g., in kg/year or absolute change), 39% (*n* = 46) examined the relationship with measurements of HGS in the follow‐up periods (e.g., in kg or weak/strong grip) and 10% (*n* = 12) reported both types of HGS outcome.

**Figure 2:**
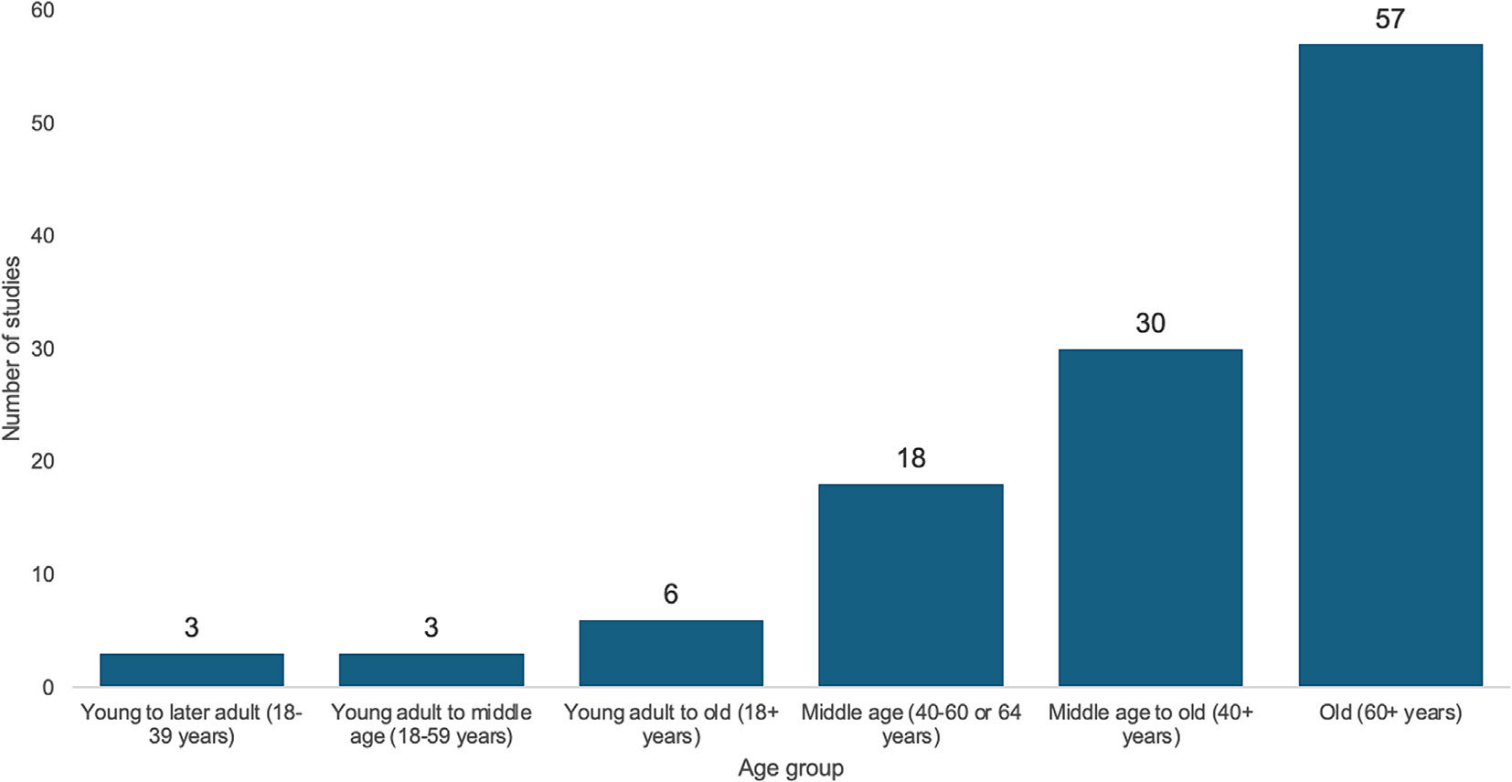
Number of studies by age group of the sample.

Studies were published between 1990 and 2022, with over 65% of all studies published in the previous 10 years. More than half of the studies were from Europe, 26% from North America, 15% from Asia, 3% from Oceania and 3% from South America. The age distribution of study samples by groups is described in *Figure* *2*, and the median age was 66 (IQR: 58.4–74.8). Sample sizes ranged from 563 to 144 369 individuals, with a median of 1904 (IQR: 925–5,131). Eight had female‐only and another 10 studies had male‐only samples, totalling 15.4% of all studies.

**Figure 3:**
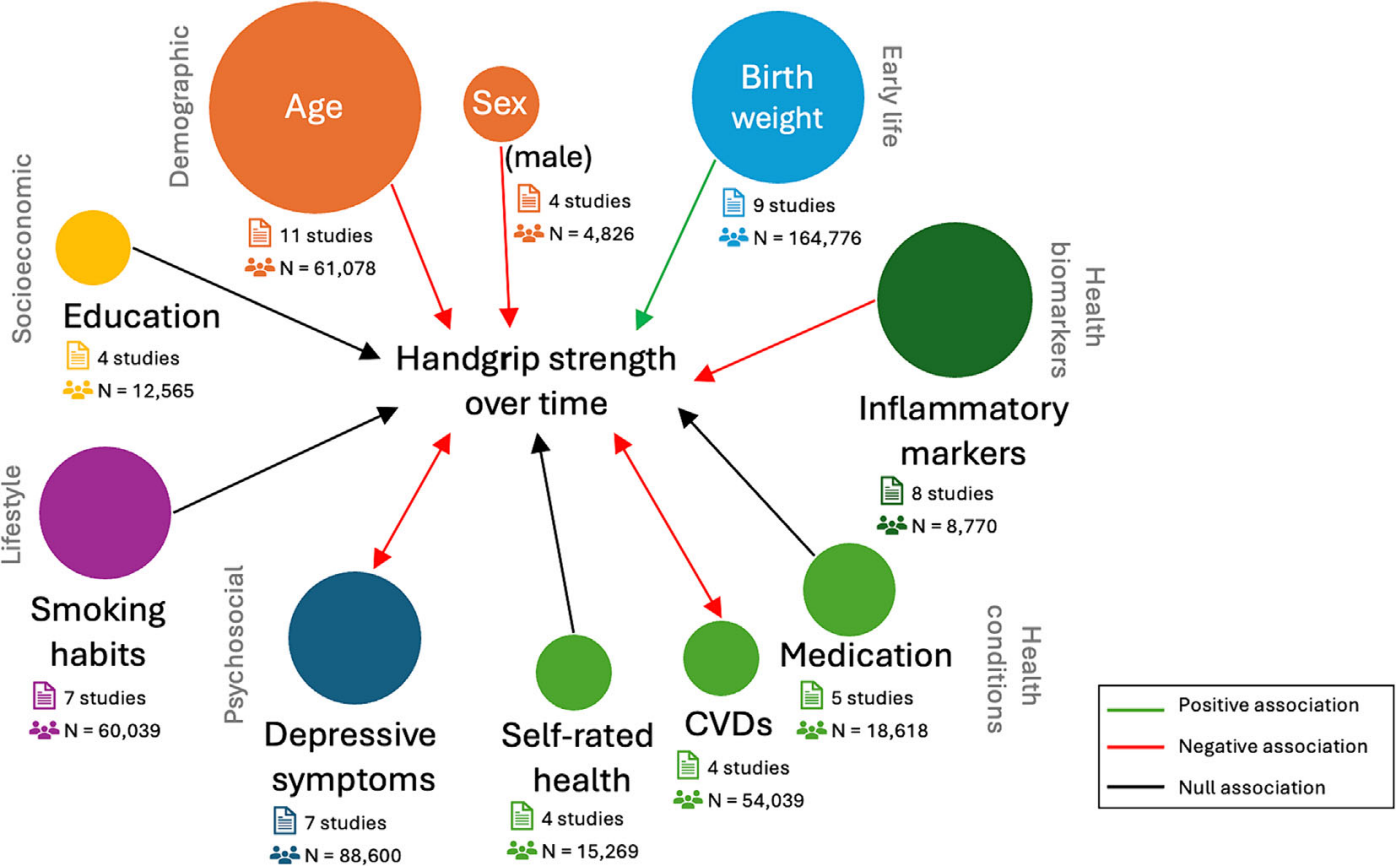
Network illustration indicating the number of studies in each arm (size of circles) and the direction of longitudinal associations with handgrip strength that were consistent (i.e., at least four studies with 60% agreement). Double‐ended arrows indicate findings of bidirectional associations. It is important to note that possible bidirectional associations are not excluded in other cases but were simply not discussed in the studies included.

A total of 103 factors were identified and allocated to the 11 domains accordingly. Each study analysed from 1 up to 18 factors as predictors of HGS over time. The variables in each of the major predictor domains and the number of studies per variable (*n*) are outlined below. *Figure* *3* shows factors in which the same direction of association with HGS over time was found in at least four articles and 60% of all studies that looked at that variable and, thus, were deemed as consistently associated with HGS. To summarize the results, we will discuss in more detail the variables that had associations consistently documented in studies. A summary of the consistent associations with the rate of change in HGS and HGS at follow‐up(s) can be found in *Tables* *1* and *2*, respectively. The list of all identified factors divided by predictors domains, the number of studies in each arm and a summary of associations with the rate of change in HGS and HGS at follow‐up(s) can be found in Appendix S1B, *Table S1* *B.1*.

**Table 1:** Summary of findings of factors that had consistent associations with the rate of change in handgrip strength

Exposure	Positive association (+)		Negative association (−)		No association (0)		Summary^b^
	Studies^a^	*N* (%)	Studies^a^	*N* (%)	Studies^a^	*N* (%)	
Demographic factors							
Age	(n/a)	0	^21^, ^22^, ^23^, ^24^, ^25^, ^26^II^27^I^28^I^29^I	9 (81.8%)	^30^II^31^	2 (19.2%)	−−
Sex (male)	(n/a)	0	^21^, ^25^, ^32^, ^33^	4 (100%)	(n/a)	0	−−
Socioeconomic factors							
Education	(n/a)	0	(n/a)	0	^21^, ^22^, ^26^, ^34^II	4 (100%)	00
Health marker/biomarker factors							
Inflammatory markers	(n/a)	0	^35^I^36^, ^37^, ^38^, ^39^F^40^	6 (75%)	^41^, ^42^I	2 (25%)	−−
Health condition factors							
Cardiovascular diseases	(n/a)	0	^21^, ^25^, ^29^, ^43^I	4 (100%)	(n/a)	0	−−
Medication use	(n/a)	0	^44^	1 (20%)	^45^, ^46^, ^47^II^26^II	4 (80%)	00
Self‐rated health	(n/a)	0	(n/a)	0	^23^, ^26^, ^32^, ^34^II	4 (100%)	00

^a^ Numbers in the columns refer to numbers in the References. If the direction of the association was specific to male or female participants of the study, ‘M’ or ‘F’ is indicated. ‘I’ and ‘II’ indicate 100% male or female sample, respectively.

^b^ Summary of association with handgrip strength (HGS): ++, repeatedly documented positive association with HGS (≥60% and at least 4 studies); 00, repeatedly documented lack of association with HGS; −−, repeatedly documented negative association with HGS; mixed, repeatedly documented inconsistent or indeterminate direction of association (e.g., non‐linear relationships, sex or age‐specific relationships, or variety of exposure variables that do not allow one conclusion).

**Table 2:** Summary of findings of factors that had consistent associations with handgrip strength at follow‐up(s)

Exposure	Positive association (+)		Negative association (−)		No association (0)		Summary^b^
	Studies^a^	*N* (%)	Studies^a^	*N* (%)	Studies^a^	*N* (%)	
Early life factors							
Birth weight	^48^, ^49^, ^50^, ^51^, ^52^, ^53^, ^54^I^55^M	8 (88.9%)	(n/a)	0	^56^	1 (11.1%)	++
Psychosocial factors							
Depressive symptoms	(n/a)	0	^22^, ^57^, ^58^, ^59^, ^60^, ^61^, ^62^F	7 (100%)	(n/a)	0	−
Lifestyle factors							
Smoking habits (current smoker)	^43^	1 (14.3%)	^49^M	1 (14.3%)	^22^, ^63^, ^64^, ^65^, ^66^II	5 (71.4%)	00

^a^ Numbers in the columns refer to numbers in the References. If the direction of the association was specific to male or female participants of the study, ‘M’ or ‘F’ is indicated. ‘I’ and ‘II’ indicate 100% male or female sample, respectively.

^b^ Summary of association with handgrip strength (HGS): ++, repeatedly documented positive association with HGS (≥60% and at least 4 studies); 00, repeatedly documented lack of association with HGS; −−, repeatedly documented negative association with HGS; mixed, repeatedly documented inconsistent or indeterminate direction of association (e.g., non‐linear relationships, sex or age‐specific relationships, or variety of exposure variables that do not allow one conclusion).

Demographic variables included age (*n* = 13), sex (*n* = 5), birth cohort (*n* = 4), marital status (*n* = 3), marital status history (*n* = 1), race/ethnicity (*n* = 1) and years to death (*n* = 1). Age and sex were the most consistent demographic‐associated factors in this review.

In almost 82% of the studies, the HGS decline became progressively steeper with age, particularly after the fifth decade of life.^27^, ^28^ In young adulthood, the annual rate of change of maximal HGS was −0.4 to −0.2 kg/year,^21^ compared with −0.6 to −0.2 kg/year in middle age and −1.0 to −0.4 kg/year in old age.^21^, ^22^, ^29^ Overall, each 1 year increase in age after the fifth decade of life was associated with a 0.03 to 0.4 kg decrease in HGS.^23^, ^24^, ^67^

In total, four studies (100%) agree with a negative association between male sex and decline in HGS. Men showed a steeper HGS decline than women in three studies, despite being around 28–47% stronger at baseline and still 21–49% stronger at follow‐up.^21^, ^32^, ^33^ The average annual rate of change in maximal HGS varied between −1.2 and −0.4 kg/year among males against −0.4 to −0.2 kg/year among females.^21^, ^33^ Moreover, men from the African American Health project showed a 99% increased risk of losing 5 kg or more in HGS over a 3 year follow‐up compared with women.^25^

The socioeconomic variables identified in studies were education (*n* = 6), occupation class (*n* = 3), income (*n* = 2), housing tenure (*n* = 1), cohabitation history (*n* = 1), employment status history (*n* = 1), country inequality history (*n* = 1) and health insurance/costs (*n* = 1).

Four studies examining the rate of decline in HGS did not find any differences based on education level, whether measured in continuous years^26^, ^34^ or categorized.^21^, ^22^ There was an indication of a cross‐sectional association between higher education and stronger grip, which may be more pronounced in women than in men.^22^, ^34^ In women from the British National Survey of Health and Development and the Swedish Adoption/Twin Study of Aging, higher education level was associated with 0.38 and a 2.20 kg stronger grip at baseline, respectively, but associations were not significant among men.^22^ Additionally, there may be a mediating effect of body weight and percentage of body fat for both sexes.^34^

Genetic factors were ageing markers (*n* = 5) and APOE alleles (*n* = 2). None of those factors had consistent associations with HGS at follow‐up or its decline.

Early life factors included birth weight (*n* = 9), growth trajectories (*n* = 5), pubertal timing (*n* = 1), childhood socioeconomic status (*n* = 3), childhood cognition (*n* = 2), motor development (*n* = 2), gestational age (*n* = 1), infant feeding (*n* = 1), adverse childhood events (*n* = 1) and childhood health biomarkers (*n* = 1).

Increased birth weight was associated with stronger grip strength at follow‐up in nine studies (81.8%), of which one study found it to be the case in males but not in females.^55^ Estimates of the linear association between birth weight *z*‐score and HGS in adulthood varied from 0.68 [95% confidence interval (CI): 0.45, 0.92] in a sample of 4304 people from Finland aged 31 years^54^ to 1.80 (95% CI: 1.75, 1.86) in 144 368 Swedish individuals with an average age of 18.3 years.^48^

Body composition factors included body mass index (BMI, *n* = 11) and BMI history (*n* = 1), lean mass (*n* = 3) and fat mass parameters (*n* = 3), height (*n* = 3) and height history (*n* = 1), weight (*n* = 2) and weight history (*n* = 3), and waist circumference (*n* = 2). None of these factors were consistently associated with HGS over time. Despite the number of studies examining BMI, findings were mixed, and in some cases, the associations were restricted to males.^49^, ^68^, ^69^

Health markers and biomarkers included inflammatory markers (*n* = 9), anti‐inflammatory markers (*n* = 9), blood pressure (*n* = 3), serum vitamin D (*n* = 3), metabolic syndrome components (*n* = 2), activities of daily living and instrumental activities of daily living disabilities (*n* = 1), fitness tests (*n* = 1), thyroid function (*n* = 1) and parathyroid function markers (*n* = 1) and plasma dp‐ucMGP (*n* = 1).

Higher concentrations of inflammatory markers were associated with a steeper decline in strength in 75% of studies. However, results must be taken with caution due to the heterogeneity of factors analysed, including interleukins (IL‐1β, IL‐1RA, IL‐6, IL‐8 and IL‐10), cytokines [tumour necrosis factor‐alpha (TNF‐α) and its receptor 1 (TNF‐αR1), C‐reactive protein (CRP) and high‐sensitivity CRP (hsCRP)], cortisol, homocysteine, serum albumin, serum haemoglobin, cholesterol, antichymotrypsin and adiponectin to leptin ratio. Among 716 older adults from the InCHIANTI study, a progressively greater rate of decline in HGS was observed with the increasing number of catabolic biomarkers, including hs‐CRP, IL‐6, IL‐1RA and TNF‐αR1. The average change was almost 6 times higher among those with four catabolic biomarkers in the highest tertile compared with those with none (−0.41 kg/year against −0.07 kg/year, respectively).^36^

Health condition factors included comorbidities (*n* = 6), diabetes mellitus (*n* = 6), cardiovascular diseases (*n* = 5), medication use (*n* = 5), self‐rated health (*n* = 4), arthritis (*n* = 3), hypertension (*n* = 3), cancer (*n* = 3), respiratory conditions (*n* = 3), pain (*n* = 2), fatigue (*n* = 2), eye conditions (*n* = 2), kyphosis (*n* = 1), metabolic syndrome (*n* = 1), hepatic conditions (*n* = 1) and other health condition categories (*n* = 1).

Four studies (100%) reported an increased risk of decline in HGS among individuals with cardiovascular diseases (CVDs). Findings also suggest a two‐way relationship between poor HGS and CVDs.^21^, ^43^ However, there were slightly different definitions of CVDs, which included one or more of the following conditions: coronary heart disease, cerebrovascular disease, peripheral vascular (or arterial) disease, angina, arrhythmia, valvular heart disease and congenital heart disease, limiting the analysis of effect sizes. As an example, findings from the 44 315 UK Biobank participants suggest a 7% increased risk of decline and a 22% increased risk of stable low HGS (< −1 *SD* of the age and sex‐specific mean) over time.^43^

Four out of five studies (80%) found no associations between HGS decline and medication use. Nevertheless, the medications varied between studies, including statin and ACE inhibitors (*n* = 2), insulin or oral anti‐diabetic medication (*n* = 1), calcium supplement and calcium channel blockers (*n* = 1), drugs with anticholinergic and sedative effects (*n* = 1) and a combination of oestrogen, thyroid hormone and corticosteroids (*n* = 1).

Even though two studies found a 0.6 to 0.9 kg stronger grip at baseline associated with better self‐rated health in older adults,^23^, ^32^ none of the four studies found longitudinal associations between those variables.

Psychosocial factors included depressive symptoms (*n* = 9), cognitive function (*n* = 5), psychiatric disorders (*n* = 2), stress (*n* = 1), life satisfaction (*n* = 1), purpose of life (*n* = 1) and partner's beliefs about ageing (*n* = 1).

There was a consistent association between depressive symptoms and weaker grip strength at follow‐up, supported by all seven studies examining this outcome (although one was a female‐specific association). The inverse association was also true, where those with weakness had an increased risk of depression than non‐weak participants.^57^, ^58^ The bidirectional relationship strongly supports the need for early detection and intervention strategies for both health outcomes to achieve reciprocal benefits.^57^, ^58^ Another interesting finding was an interplay between BMI, depressive symptoms and decreased grip strength shown by Luo et al.^57^ and Rantanen et al.^69^

Lifestyle factors were the most extensively studied associated factors in this review. Exposure variables analysed were physical activity (*n* = 14), smoking habits (*n* = 12), alcohol consumption (*n* = 7), protein intake (*n* = 7), physical inactivity or sedentarism (*n* = 6), fruit and vegetable intake (*n* = 5), physical activity history (*n* = 3), occupational physical activity (*n* = 3), healthy habits (*n* = 3), fat intake (*n* = 2), red and processed meat intake (*n* = 2), Mediterranean diet (*n* = 2), energy intake (*n* = 1), carbohydrate intake (*n* = 1), oily fish intake (*n* = 1), ultra‐processed food intake (*n* = 1), cereals, low‐fat milk and fish intake (*n* = 1), selenium intake (*n* = 1), antioxidants intake (*n* = 1), dietary patterns (*n* = 1) and variety (*n* = 1), DASH diet (*n* = 1), Japanese Food Guide Spinning Top (JFG‐ST) diet (*n* = 1), Nordic diet (*n* = 1), healthy diet index (*n* = 1) and sleep patterns (*n* = 1).

Despite the many studies in this domain (38 in total), only smoking habits had consistent results with follow‐up measurements of HGS, pointing to a lack of association between those variables. Nonetheless, smoking has been extensively associated with adverse health outcomes such as an increased risk of CVDs, negative changes in the circulatory system and oxidative stress, which may end up negatively affecting the musculoskeletal system.^21^, ^22^ The effects of physical activity (PA) were the most frequently analysed lifestyle factors in this review. However, the results were mixed, as were the measures used in studies that looked at levels of PA, inactivity/sedentary lifestyle, work‐related PA and/or changes in PA.

Reproductive factors included the following variables: sex hormone levels including testosterone (*n* = 2), menopause (*n* = 2), age at hysterectomy (*n* = 1), gynaecological/breast conditions (*n* = 1), hormone replacement therapy (*n* = 1) and lactation duration across pregnancies (*n* = 1). Due to the small number of studies in this domain, no consistent association was found.

Environmental factors included exposure to natural environments (*n* = 1) and occupational exposure to chemicals (*n* = 1). The lack of studies in this area prevented drawing any further conclusions.

## Discussion

Findings from this review suggest that factors associated with stronger HGS measurements over time include higher birth weight whereas depressive symptoms in adulthood were consistently associated with weaker grip. Surprisingly, there was no association between current or past smoking and HGS. Increased age, male sex, cardiovascular diseases and higher levels of inflammatory markers were repeatedly associated with a steeper decline in HGS. Finally, education level, medication use and self‐rated health were not associated with the change in grip.

Low HGS has been linked to premature mortality and a wide range of health conditions during ageing, yet little is known about the mechanisms determining weakness to date.^3^ To the best of our knowledge, this is the first study to systematically and comprehensively review the evidence for predictors of HGS from longitudinal studies. Determinants research can be used to identify high‐risk groups, thus supporting better decision‐making and targeted interventions.^18^, ^70^ Determinants such as age and sex are not modifiable; however, there were several modifiable determinants of HGS identified in this review, which can be used to guide health interventions from early life to adulthood.^70^ Consistent associations with inflammatory markers, cardiovascular diseases and depressive symptoms suggest those factors should be prioritized in interventions. Additionally, the bidirectional associations with mental health and cardiovascular diseases indicate that joint efforts to tackle mental and physical health declines can produce mutual benefits.^21^, ^43^, ^57^, ^58^, ^71^ This review also sheds light on gaps in the literature, particularly genetics, reproductive and environmental factors, given the small number of studies in those domains.

The results from this review seem to agree with the conceptual model proposed by McGrath et al.,^3^ where metabolic and neurological systems drive the associations between HGS and health outcomes. Some of the included studies showed parallel associations between HGS and depression^57^, ^58^ and cardiovascular diseases,^21^, ^43^ indicating that there likely are common mediating factors affecting nervous and muscular systems function and, thereby, muscle strength and health.^3^ Shared aetiological factors such as ageing and inflammation may help to better understand the aetiology of weakness.^3^, ^72^

All the 11 major determinants identified in this study seem to have some kind of association with HGS over time. It is noteworthy, however, that only about 10% of the 103 exposure variables had consistent associations with HGS over time reported in studies. Between 21% and 35% of the variables did not show any association with HGS follow‐up measurements or the rate of decline, but results, as well as methodological approaches, were mostly not uniform, and thus, those variables should not be dismissed. The same applies to the 8–21% that showed weak evidence of positive associations and the 17–29% that showed negative associations, that is, more research is needed to confirm those results. Lastly, 24–32% of variables had associations deemed as inconsistent or indeterminate, mainly due to sex‐graded or non‐linear associations.

Interestingly, there was no consensus regarding the association between PA and HGS over time, despite being the most extensively studied associated factor in this review. A systematic review of cross‐sectional studies showed that the practice of regular mild‐to‐moderate or moderate‐to‐vigorous PA was associated with greater HGS levels.^18^ In this review, 57.1% of studies found a positive association between higher PA levels and follow‐up measurements. However, there seemed to be a lack of association with the rate of decline in HGS (in 44.4% of studies). Similar findings were reported in studies from Australia and the Netherlands, which analysed the course of physical functioning over time using both subjective and objective measures of muscle function and performance.^73^, ^74^ Nevertheless, in the Dutch study, those who remained inactive through midlife to older ages (40–55+ years) showed 1.5–1.6 higher odds of physical decline than those who were physically active in one or both of those periods.^74^ As argued by Peeters et al.,^73^ cumulative activity, rather than activity at any given time, may have a greater impact on physical functioning, considering the fluctuations in PA levels across the lifespan. In this review, few studies looked at PA history (*n* = 3), using different measurement methods, which limits the interpretation of findings. Nonetheless, evidence suggests that PA type, intensity, frequency and duration are other important factors to consider in this relationship.^21^, ^75^, ^76^

A common finding across themes was that researchers could generally observe cross‐sectional associations with HGS but not longitudinal associations with HGS change. As an example, there was weak evidence of a non‐association of height with the rate of change, even though height is a widely recognized influencing factor of HGS.^8^, ^77^ Such findings might suggest that the rate of decline is more linked to age and sex than any other factors. Associations with age and sex were more consistent; however, they also depended on the follow‐up period and the age range of the sample.^25^, ^30^, ^31^ Because HGS remains fairly stable in shorter periods, especially between the second and the fifth decade of life, those who followed participants for shorter periods would have not been able to evaluate the associations effectively.^8^, ^78^

This review highlights that the rate of decline in HGS varies notably across age groups. Establishing norms for HGS or identifying consistent patterns across populations is challenging due to variations in other potential influencing factors such as sex, region, health conditions and levels of physical activity across different studies.^79^ The European Working Group on Sarcopenia in Older People reported a variation in loss of strength in the order of 1.5–5% from the age of 50 years onwards.^78^ Studies included in this review show a variation of 0.03–0.4 kg decrease in strength for each year above the fifth decade.^23^, ^24^, ^67^ Although this effect may be viewed as insignificant (<5–6.5 kg difference), there is currently no consensus regarding the minimal clinically important difference in HGS.^80^ Furthermore, the accumulation of continuous loss over the years can become significant.

While everyone will decline in strength with age, those who maintained a higher measurement over time are likely to remain stronger than those who had weaker grip strength.^28^, ^73^ As an example, findings from this review suggest that while males may exhibit a more pronounced decline in HGS, they still maintain a significant strength advantage over females. Females are at higher risk of developing musculoskeletal disorders later in life than males.^11^ Therefore, ensuring optimal muscle strength as early as possible is crucial. Positive results found between better parameters in early life, such as birth weight and stronger grip strength point out that interventions should cover much younger ages. However, interventions for improving muscle strength (as well as muscle strength research, as demonstrated in this review) are currently focused on older populations.^73^

This is the first systematic summary of the predictive factors of HGS. We reviewed 117 studies assessing longitudinal factors associated with HGS. Despite the careful search strategy construction, some studies may have been missed. Another limitation of this review is its semiquantitative design, which is based on previously established definitions of consistency of results that are, nonetheless, open to interpretation and debate.^18^, ^19^, ^20^ The comparison between studies is challenging due to the varied age range and sex distribution of the samples, differences in protocols of HGS measurement (equipment used, type of HGS measure, hand(s) measured, units of measure), and covariates used in analyses (refer to Appendix SB, *Table S* *B.2*). For instance, HGS tends to increase with the number of trials, as participants become more familiar with the dynamometer. Even within multiple attempts, the maximal HGS will be higher than the average HGS.^35^, ^79^ In addition, maximal HGS is approximately 10% higher in the dominant hand than the non‐dominant hand.^35^ Finally, comparing results between studies is limited by other factors such as the inter‐instrument reliability of dynamometers.^8^, ^35^ Those issues have prompted several authors to advocate for a standardized approach.^35^, ^37^, ^79^ A review of measurement of grip strength has recommended instruments such as the Jamar dynamometer, the Baltimore Therapeutic Equipment (BTE) Work Simulator, the BTE Primus, and the Martin Vigorimeter. These instruments have acceptable validity and reliability but use different units of measurement, a consideration when assessing measures against normative standards.^35^ Proposed protocols also emphasize recording hand dominance, conducting multiple measurements on each hand and recording maximal HGS as the greatest grip force generated across all trials.^79^

As also observed in previous systematic reviews about HGS, the wide range of exposure variables and many differences in how those variables were measured and analysed against HGS precludes the use of statistical techniques to estimate meaningful and valid pooled estimates of the associations between exposure and outcome.^37^ The mixed findings or weak evidence for most of the associations only highlight the methodological issues. As stated by McGrath,^79^ ‘A consensus statement for better standardising HGS protocols is warranted to decrease internal threats to validity, create standardization in assessments, and allow for comparisons of HGS findings across investigations’.

## Conclusions

This semiquantitative review highlighted evidence for associations between various demographic, socioeconomic, genetic, early life, body composition, health markers/biomarkers, health condition, psychosocial, lifestyle, reproductive and environmental factors with HGS. Our results confirm the complexity of processes contributing to muscle strength capacity. It may be that age and sex are the main drivers of the decline in strength overall, but there are other factors contributing to this phenomenon, including birth weight, depression, inflammation and cardiovascular factors.

Several insights for future research can be derived from the present findings. Even among variables that have been repeatedly analysed in studies, we were not able to analyse effect sizes due to methodological heterogeneity, which limits the practical application of findings. Furthermore, many variables have been understudied and, therefore, should be emphasized in future studies, especially modifiable factors that are related to the development of chronic diseases, such as body fat, diet, sleep and stress. Other modifiable factors that showed indeterminate associations with HGS over time, such as physical activity and BMI, also warrant further investigation and standardization of analysis. As these factors tend to change over time, it would be interesting to look at historical exposure and its effect on HGS rather than looking at time‐specific exposure. Ultimately, this area of research should give greater focus to younger populations, before the fifth decade of age. Identifying what determines better muscle strength before the decline leads off is crucial for improving intervention and prevention strategies. To overcome those challenges and improve the quality of evidence, there is a need for collaboration and consensus among researchers, health practitioners and stakeholders on standardized protocols for HGS testing and reporting.

## Funding

G. D. M. is an NHMRC Leadership Fellow (grant number APP2009577). G. I. M. is supported by a National Health and Medical Research Council Investigator Grant (grant number APP2008702). L. W. R. and S. B. are PhD student recipients of UQ Research Training Stipend Earmarked scholarships.

## Supporting information


**Appendix S1.** Supporting Information
