# Supplementary material for: Factors associated with handgrip strength across the life course: A systematic review
Source: J Cachexia Sarcopenia Muscle. 2024 Aug 26;15(6):2270–80. doi: 10.1002/jcsm.13586 (PMC11634504; doi:10.1002/jcsm.13586)
Supplement: Supplementary file 1 — Appendix S1. Supporting Information [file JCSM-15-2270-s001.docx]

**The correlates of handgrip strength across the life course: A systematic review**

**Appendices**

# Appendix A - Search Strategies

## A.1. MEDLINE via Ebsco

(( (TI (("handgrip strength" OR "hand grip strength" OR "grip strength" OR "muscle strength" OR "musculoskeletal development")) OR AB (("handgrip strength" OR "hand grip strength" OR "grip strength" OR "muscle strength" OR "musculoskeletal development")) )

N3 (TI (("influence" OR "predictor*" OR "correlates" OR "determinant*" OR "modifiable factor*" OR "association*" OR "life course" OR "lifespan" OR "birth")) OR AB (("influence" OR "predictor*" OR "correlates" OR "determinant*" OR "modifiable factor*" OR "association*" OR "life course" OR "lifespan" OR "birth")) ) ) OR ( ((TI ( ("influence" OR "predictor*" OR "correlates" OR "determinant*" OR "modifiable factor*" OR "association*" OR "life course" OR "lifespan" OR "birth") ) OR AB ( ("influence" OR "predictor*" OR "correlates" OR "determinant*" OR "modifiable factor*" OR "association*" OR "life course" OR "lifespan" OR "birth") )) AND (MM "Muscle Strength" OR MM "Hand Strength")) ) ) AND ( (MH "Epidemiologic Studies" OR MH "Case-Control Studies+" OR MH "Cohort Studies+"OR MH "Cross-Sectional Studies" OR PT Comparative Study OR PT Review OR “follow-up” OR "case control" OR "cohort study" OR "Cohort studies" OR "cohort analy*" OR "follow up study" OR "follow up studies" OR "observational study" OR "observational studies" OR "longitudinal" OR "retrospective" OR "population-based" OR "review") )

## A.2. Embase via Elsevier

((('handgrip strength' OR 'grip strength' OR 'muscle strength' OR 'musculoskeletal development') NEAR/3 ('influence' OR 'predictor*' OR 'correlates' OR 'determinant*' OR 'modifiable factor*' OR 'association*' OR 'life course' OR 'lifespan' OR 'birth')) OR (('influence' OR 'predictor*' OR 'correlates' OR 'determinant*' OR 'modifiable factor*' OR 'association*' OR 'life course' OR 'lifespan' OR 'birth') AND ('muscle strength'/mj OR 'hand strength'/mj))) AND ('epidemiology'/mj OR 'case control study'/exp OR 'cohort analysis'/exp OR 'cross-sectional study'/exp OR 'comparative study'/exp OR 'review'/exp OR 'follow-up' OR 'case control' OR 'cohort study' OR 'cohort studies' OR 'cohort analy*' OR 'follow up study' OR 'follow up studies' OR 'observational study' OR 'observational studies' OR 'longitudinal' OR 'retrospective' OR 'population-based' OR 'review') AND [embase]/lim AND ([article]/lim OR [article in press]/lim OR [review]/lim)

## A.3. SPORTDiscus

(((TI ( ("handgrip strength" OR "hand grip strength" OR "grip strength" OR "muscle strength" OR "musculoskeletal development") ) OR AB ( ("handgrip strength" OR "hand grip strength" OR "grip strength" OR "muscle strength" OR "musculoskeletal development")) ) N3 (TI ( ("influence" OR "predictor*" OR "correlates" OR "determinant*" OR "modifiable factor*" OR "association*" OR "life course" OR "lifespan" OR "birth") ) OR AB ( ("influence" OR "predictor*" OR "correlates" OR "determinant*" OR "modifiable factor*" OR "association*" OR "life course" OR "lifespan" OR "birth") ) )) OR ((TI ( ("influence" OR "predictor*" OR "correlates" OR "determinant*" OR "modifiable factor*" OR "association*" OR "life course" OR "lifespan" OR "birth") ) OR AB ( ("influence" OR "predictor*" OR "correlates" OR "determinant*" OR "modifiable factor*" OR "association*" OR "life course" OR "lifespan" OR "birth") )) AND (DE "GRIP strength" OR DE "MUSCLE strength"))) AND (DE "EPIDEMIOLOGY" OR “case control stud*” OR DE "COHORT analysis" OR “cross-sectional stud*” OR “comparative study” OR “follow-up” OR "case control" OR "cohort study" OR "Cohort studies" OR "cohort analy*" OR "follow up study" OR "follow up studies" OR "observational study" OR "observational studies" OR "longitudinal" OR "retrospective" OR "population-based" OR "review")

# Appendix B – Supplementary tables

### Supplementary Table B.1. Summary of findings the associations between potential predictors and handgrip strength by predictors domains

| Potential predictor factors by domains | Total number of studies | Associations with the rate of change in HGS^a^ | Associations with HGS at follow-up(s)^a^ | References |  |
| --- | --- | --- | --- | --- | --- |
| Demographic factors |  |  |  |  |  |
| Age | 11 | -- | - | (1-13) |  |
| Sex (male) | 5 | -- | + | (6, 9, 11, 14, 15) |  |
| Marital status (married) | 4 | 0 | (n/a) | (12-14) |  |
| Marital status history (never married) | 3 | (n/a) | - | (16) |  |
| Birth cohort (younger cohort) | 1 | ? | + | (1, 7, 17, 18) |  |
| Race/Ethnicity (nonwhite) | 1 | ? | (n/a) | (5) |  |
| Years to death | 1 | + | (n/a) | (8) |  |
| Socioeconomic factors |  |  |  |  |  |
| Education | 6 | 00 | + | (2, 9, 11, 12, 19, 20) |  |
| Occupation class (manual) | 3 | 0 | - (13) | (14, 20) |  |
| Employment status history (continuously working) | 2 | (n/a) | + | (21) |  |
| Income | 1 | 0 | (n/a) | (5, 12) |  |
| Housing tenure (not owned/mortgaged) | 1 | 0 | (n/a) | (13) |  |
| Cohabitation history (continuously living with partner) | 1 | (n/a) | + | (16) |  |
| Country inequality history (higher inequality) | 1 | (n/a) | - | (22) |  |
| Health insurance/costs | 1 | ? | (n/a) | (6) |  |
| Genetic factors |  |  |  |  |  |
| Aging markers | 5 | ? | ? | (23-26) |  |
| APOE alleles | 2 | ? | (n/a) | (27, 28) |  |
| Early life factors |  |  |  |  |  |
| Birth weight | 9 | 0 | ++ | (17, 20, 29-35) |  |
| Growth trajectories (higher weight/height gain in childhood) | 5 | (n/a) | ? | (20, 30, 32, 34, 36) |  |
| Childhood SES (classes IV and V) | 3 | ? | ? | (12, 20, 37) |  |
| Childhood cognition | 2 | 0 | ? | (20, 38) |  |
| Motor development (earlier development) | 2 | (n/a) | ? | (20, 32) |  |
| Pubertal timing (later maturation) | 1 | (n/a) | ? | (20) |  |
| Gestational age | 1 | (n/a) | 0 | (30) |  |
| Infant feeding (breastfed only) | 1 | (n/a) | ? | (34) |  |
| Adverse childhood events | 1 | (n/a) | ? | (39) |  |
| Childhood health biomarkers (CVD risk factors) | 1 | (n/a) | - | (36) |  |
| Body composition factors |  |  |  |  |  |
| BMI | 11 | ? | + | (5, 6, 9, 11, 13, 14, 20, 40-43) |  |
| Lean mass parameters | 3 | ? | (n/a) | (3, 4, 44) |  |
| Fat mass parameters | 3 | 0 | (n/a) | (13, 19, 44) |  |
| Height | 3 | 0 | + | (8, 13, 14) |  |
| Weight history (weight loss) | 3 | - | (n/a) | (2, 10, 11) |  |
| Weight | 2 | 0 | (n/a) | (8, 13) |  |
| Waist circumference | 2 | ? | - | (40, 45) |  |
| BMI history (greater gains) | 1 | (n/a) | 0 | (46) |  |
| Height history (height loss) | 1 | ? | (n/a) | (2) |  |
| Health marker/biomarker factors |  |  |  |  |  |
| Inflammatory markers | 9 | -- | 0 | (47-55) |  |
| Anti-inflammatory markers | 9 | ? | + | (49, 53, 56-62) |  |
| Blood pressure | 3 | ? | 0 | (6, 12, 45) |  |
| Serum vitamin D | 3 | + | (n/a) | (63-65) |  |
| Metabolic Syndrome components | 2 | ? | 0 | (19, 45) |  |
| ADL and IADL disabilities | 1 | 0 | (n/a) | (6) |  |
| Fitness tests | 1 | 0 | (n/a) | (66) |  |
| Thyroid function (high T3, T4, TSH) | 1 | ? | (n/a) | (67) |  |
| Parathyroid function (high PTH) | 1 | - | (n/a) | (65) |  |
| Plasma dp-ucMGP | 1 | (n/a) | - | (68) |  |
| Health condition factors |  |  |  |  |  |
| Number of comorbidities | | 6 | ? | - | (2, 11, 13, 20, 41, 69) |
| Diabetes | | 6 | - | - | (3, 9-11, 70, 71) |
| Cardiovascular diseases | 5 | -- | - | (6, 9-11, 41) |  |
| Medication use | 5 | 00 | (n/a) | (2, 70, 72-74) |  |
| Self-rated health | 4 | 00 | (n/a) | (2, 13, 14, 19) |  |
| Cancer | 3 | 0 | - | (3, 10, 41) |  |
| Hypertension | 3 | ? | (n/a) | (3, 10, 11) |  |
| Arthritis | 3 | 0 | (n/a) | (10, 11, 14) |  |
| Metabolic syndrome | 1 | (n/a) | ? | (45) |  |
| Kyphosis | 1 | 0 | (n/a) | (75) |  |
| Respiratory conditions | 1 | - | (n/a) | (10, 11, 41) |  |
| Eye conditions | 1 | - | 0 | (41, 76) |  |
| Hepatic conditions | 1 | (n/a) | - | (77) |  |
| Other health conditions categories | 1 | ? | ? | (41) |  |
| Pain | 1 | - | (n/a) | (6, 11) |  |
| Fatigue | 1 | - | - | (78, 79) |  |
| Psychosocial factors |  |  |  |  |  |
| Depressive symptoms | 9 | -- | -- | (9, 12, 14, 42, 80-84) |  |
| Cognitive function | 5 | + | + | (9, 20, 82, 85, 86) |  |
| Psychiatric disorders | 2 | 0 | - | (11, 41) |  |
| Life satisfaction | 1 | (n/a) | + | (82) |  |
| Purpose of life | 1 | + | 0 | (87) |  |
| Partner's beliefs about ageing (positive beliefs) | 1 | (n/a) | + | (88) |  |
| Stress | 1 | ? | ? | (12) |  |
| Lifestyle factors |  |  |  |  |  |
| Physical activity | 14 | ? | + | (3, 5, 6, 11, 12, 14, 17, 20, 41, 89-93) |  |
| Smoking habits (current smoker) | 12 | 0 | 00 | (2, 5, 11-13, 17, 20, 41, 91, 93-95) |  |
| Alcohol consumption (current drinking) | 7 | 0 | ? | (2, 11, 13, 17, 91, 94, 96) |  |
| Protein intake (in weight) | 7 | ? | ? | (2, 92, 97-101) |  |
| Physical inactivity/Sedentarism | 6 | 0 | - | (2, 3, 13, 41, 43, 102) |  |
| Fruit and vegetable intake (regular/frequent) | 5 | 0 | 0 | (41, 59, 91, 94, 96) |  |
| Physical activity history (decreased activity) | 3 | - | - | (11, 94, 103) |  |
| Occupational physical activity | 3 | - | ? | (11, 12, 104) |  |
| Healthy habits (less healthy behaviour) | 3 | - | 0 | (69, 91, 93) |  |
| Red and processed meat intake (frequent) | 2 | 0 | ? | (41, 96) |  |
| Mediterranean diet | 2 | ? | (n/a) | (105, 106) |  |
| Oily fish intake (infrequent) | 1 | 0 | - | (41) |  |
| Ultra-processed food intake (in weight) | 1 | - | (n/a) | (107) |  |
| Cereals, low-fat milk, fish intake (frequent) | 1 | (n/a) | 0 | (96) |  |
| Energy intake (in kcal) | 1 | (n/a) | + | (101) |  |
| Carbohydrate intake (% energy) | 1 | (n/a) | - | (101) |  |
| Fat intake (% energy) | 1 | (n/a) | 0 | (96, 101) |  |
| Selenium intake (low intake) | 1 | 0 | (n/a) | (108) |  |
| Antioxidants intake (in weight) | 1 | + | (n/a) | (109) |  |
| Dietary patterns | 1 | ? | (n/a) | (110) |  |
| Dietary variety | 1 | + | (n/a) | (111) |  |
| DASH diet | 1 | 0 | (n/a) | (106) |  |
| Japanese Food Guide Spinning Top diet | 1 | 0 | (n/a) | (106) |  |
| Nordic diet | 1 | (n/a) | ? | (96) |  |
| Healthy diet score | 1 | (n/a) | 0 | (93) |  |
| Sleep duration | 1 | ? | (n/a) | (112) |  |
| Reproductive factors |  |  |  |  |  |
| Menopausal status | 2 | 0 | 0 | (5, 113) |  |
| Testosterone levels | 2 | ? | (n/a) | (53, 114) |  |
| Age at hysterectomy (before 40y) | 1 | (n/a) | - | (113) |  |
| Gynaecology/breast conditions | 1 | 0 | 0 | (41) |  |
| Hormone replacement therapy (HRT users) | 1 | 0 | (n/a) | (5) |  |
| Lifetime breastfeeding | 1 | (n/a) | + | (115) |  |
| Environmental factors |  |  |  |  |  |
| Proximity to green and blue spaces | 1 | ? | (n/a) | (116) |  |
| Occupational exposure to chemicals | 1 | 0 | (n/a) | (117) |  |

^a^Summary of association with handgrip strength (HGS): +, weak evidence of a positive association with HGS (≥60% studies); ++, repeatedly documented positive association with HGS (≥60% and at least 4 studies); 0, weak evidence of lack of association with HGS; 00, repeatedly documented lack of association with HGS; --, repeatedly documented negative association with HGS; ?, weak evidence of indeterminate direction of association (e.g., non-linear relationships, sex or age-specific relationships, or variety of exposure variables that don’t allow one conclusion); ??, repeatedly documented inconsistent or indeterminate direction of association.

### Supplementary Table B.2. Studies examining the longitudinal relationship between exposure variables and handgrip strength (n = 155).

| **Included papers ID (Author-year) and reference** | **Country** | **Cohort study name** | **Initial sample size** | **Age group/range** | **Gender distribution** | **Years of follow-up** | **Type of HGS measurement** | **HGS unit** | **Hand(s) measured** | **Number of HGS measurements** | **Number of exposures analysed** | **Covariates** |
| --- | --- | --- | --- | --- | --- | --- | --- | --- | --- | --- | --- | --- |
| Ahlqvist, 2019 | Sweden | Birth cohort from Sweden | 144,369 | Young adult (18-29 years) | M: 100% | 18 years | Maximal HGS (follow-up) | N | Dominant hand | 1 | 1 | Parity, maternal age, maternal diabetes, maternal hypertension, caesarean section, conscription office and highest parental education |
| Batterham, 2013 | Australia | Canberra Longitudinal Study | 590 | Old (70+ years) | F: 51% M: 49% | 12 years | Not described (decline) | kg | Both hands | 2 | 1 | Initial age, sex, education, dementia |
| Beller, 2019 | Germany | Survey of Health, Ageing and Retirement in Europe (SHARE) | 22,550 | Middle age to old (50+ years) | F: 54% M: 46% | 9 years | Maximal HGS (follow-up and decline) | kg | Both hands | ≥3 | 2 | Stratified by age, period, and birth cohort |
| Bertoni, 2018 | Italy | SHARE | 6,526 | Old (70+ years) | F: 56% M: 44% | 4 years | Maximal HGS (follow-up) | kg | Both hands | 3 | 1 | Age, sex, country, education, homeownership, income, marital status, number of children and grandchildren, health conditions, mobility limitations, smoking habits, physical activity, BMI, social activity, diet |
| Bielemann, 2016 | Brazil | 1982 Pelotas birth cohort | 3,470 | Young adult (30 years) | F: 50.1% M: 49.9% | 30 years | Maximal HGS (follow-up) | kg | Both hands | 1 | 3 | Family income at birth, maternal schooling, maternal smoking during pregnancy, maternal pre-pregnancy BMI, skin colour, birth weight, and nutritional status at 2y, height, physical activity, lean mass |
| Botoseneanu, 2015 | US | Cohort from Kusatsu, Japan | 1,381 | Old (65+ years) | F: 58% M: 42% | 8 years | Average HGS (decline) | kg | Dominant hand | ≤8 | 4 | Height, weight, smoking habits, alcohol consumption, BP, and medication use |
| Cervo, 2021 | Australia | Concord Health and Ageing in Men Project (CHAMP) | 794 | Old (70+ years) | M: 100% | 5 years | Average HGS (decline) | kg | Dominant hand | 2 | 1 | Age, country of birth, body fat percentage, physical activity, smoking status, total energy intake, dietary vitamin D and calcium intakes, vitamin D and calcium supplements use, NSAIDs and bisphosphonate use, presence of musculoskeletal disease, frailty status, and number of other comorbidities. |
| Chang, 2021 | Singapore | Singapore Chinese Health Study (SCHS) | 9,581 | Old (70+ years) | F: 57.6% M: 42.4% | 20 years (median) | Maximal HGS (follow-up) | kg | Both hands | 1 | 2 | Sex, age, BMI, smoking habits, alcohol consumption, diet (Mediterranean diet score) |
| Charles, 2006 | US | Honolulu-Asia Aging Study | 3,522 | Middle age to old (45+ years) | M: 100% | 25 years | Not described (decline) | kg | Not described | 4 | 1 | Age, BMI, physical activity, blood glucose, smoking, CASI score, education, haemoglobin level, arthritis, forearm fracture, participant's orientation, diabetes medication, manual labour, and baseline HGS |
| Cheval, 2019 | Switzerland | SHARE | 24,179 | Middle age to old (50+ years) | F: 55.7% M: 44.3% | 12 years | Average HGS (follow-up) | kg | Both hands | 6 | 1 | Childhood socioeconomic circumstances, birth cohort, attrition, childhood health problems, height, education, occupation, housing situation, unhealthy behaviours (physical activity, diet, smoking habits, alcohol consumption) |
| Cooper, 2008 | UK | Medical Research Council National Survey of Health and Development (MRC NSHD) | 1,386 | Middle age (53 years) | F: 100% | 53 years | Maximal HGS (follow-up) | kg | Both hands | 1 | 2 | Height and weight at 53y, father's occupational class at 11y, head of household occupational class at 53y, cognitive ability at 8y, smoking status at 53y, parity at 53y |
| Cooper, 2011 | UK | MRC NSHD | 2,442 | Middle age (53 years) | F: 51.3% M: 48.7% | 53 years | Maximal HGS (follow-up) | kg | Both hands | 1 | 1 | Sex, current height and weight, socioeconomic position, smoking and health problems at 53y, physical activity levels |
| Cooper, 2014 | UK | MRC NSHD | 1,511 | Old (60-64 years) | F: 51.8% M: 48.2% | 45 years | Maximal HGS (follow-up) | kg | Both hands | 1 | 1 | Sex |
| Cooper, 2016 | UK | MRC NSHD | 2,093 | Middle age (53 and 60–64 years) | F: 52.9% M: 47.1% | 7-11 years | Maximal HGS (follow-up and decline) | kg | Both hands | 2 | 2 | Sex and health indicator count or behavioural risk factor score |
| Cooper, 2017a | UK | UK Biobank study | 66,582 | Old (60+ years) | F: 49.8% M: 50.2% | 4.5 years | Maximal HGS (follow-up) | kg | Both hands | 2 | 1 | Age, sex, height, weight, overall health status, education, occupation, smoking status, Townsend deprivation index scores (SES) |
| Cooper, 2017b | UK | MRC NSHD | 1,954 | Middle age (53 and 60–64 years) | F: 53% M: 47% | 7-11 years | Maximal HGS (follow-up and decline) | kg | Both hands | 2 | 1 | Sex, birth weight, paternal occupational class, and maternal educational level, own occupational class and cumulative scores of behavioural risk and health status, own educational level, and cognitive ability at 53y |
| de Carvalho, 2019 | Brazil | English Longitudinal Study of Ageing (ELSA) | 5,181 | Middle age to old (50+ years) | F: 54.6% M: 45.4% | 8 years | Maximal HGS (decline) | kg | Dominant hand | 3 | 2 | Age, economic circumstances, health conditions, lifestyle, biomarkers, physical function, depression, and cognitive function |
| de Keijzer, 2019 | Spain | Whitehall II study | 5,759 | Old (60+ years) | F: 27.3% M: 72.7% | 11 years | Average HGS (decline) | kg | Not described | 2 | 1 | Age squared, sex, ethnicity, height, marital status, alcohol consumption, smoking status, intake of fruit and vegetables, educational attainment, employment grade, country-specific tertiles of income and employment scores, rurality |
| de Lima, 2021 | Brazil | EpiFloripa adult and EpiFloripa aging | 2,059 | Young adult to middle age (20–59 years) | F: 59.1% M: 40.9% | 5 years | Sum of maximal HGS (follow-up) | kgf | Both hands | 3 | 5 | Age, sex, schooling, and per capita family income |
| de Vries, 2013 | UK | ELSA, SHARE, U.S. Health and Retirement Study (HRS) | 38,162 | Middle age to old (50+ years) | F: 53.4% M: 46.6% | 46 years | Maximal HGS (follow-up) | kg | Dominant hand | 1 | 1 | Age, sex, education, income, wealth and multiplicative interaction between age and gender, country’s GDP, and GDP trends |
| Dodds, 2013 | UK | MRC NSHD | 1,645 | Old (60-64 years) | F: 52.7% M: 47.3% | 24-28 years | Maximal HGS (follow-up) | kg | Both hands | 1 | 1 | Sex, educational level, occupational class, smoking status, height, weight, presence of limiting disability, and LTPA levels |
| Du, 2020 | US | Bogalusa Heart Study | 718 | Young adult to middle age (35-55 years) | F: 59.5% M: 40.5% | 39.3 years (mean) | Maximal HGS (follow-up) | kg | Both hands | 1 | 2 | Adulthood age, sex, ethnicity, diagnosis of diabetes and cardiovascular disease, and antihypertensive and lipid-lowering medication use |
| Ferrari, 2020 | Germany | KORA-Age study | 731 | Old (65+ years) | F: 49.3% M: 50.7% | 3 years | Maximal HGS (decline) | kg | Both hands | 2 | 2 | Age, sex, BMI, physical activity, multimorbidity, and smoking |
| Forrest, 2007 | US | Study of Osteoporotic Fractures | 9,372 | Old (65+ years) | F: 100% | 10 years | Average HGS (decline) | kg | Both hands | 2 | 11 | Baseline age and HGS |
| Forrest, 2012 | US | Prostate Cancer Screening Study on the Caribbean Island of Tobago | 1,710 | Young adult to old (29–89 years) | M: 100% | 4.5 years | Average HGS (decline) | kg | Both hands | 2 | 7 | Age, BMI, arm lean mass, diabetes, change in arm lean mass during follow-up, baseline HGS |
| Garcia-Esquinas, 2021 | Spain | Seniors-ENRICA-2 cohort | 2,548 | Old (65+ years) | F: 52.9% M: 47.1% | 2 years | Sum of maximal HGS (follow-up) | kg | Both hands | 2 | 1 | Age, sex, education, BMI, diet quality, protein intake, number of vitamins with intake above RDA, physical activity, smoking status, alcohol consumption, and comorbidities |
| Granic, 2016a | UK | The Newcastle 85+ Study | 813 | Old (85+ years) | F: 61.5% M: 38.5% | 5 years | Average HGS (decline) | kg | Both hands | 5 | 9 | Sex, education, occupational class, marital status, physical activity, smoking, alcohol intake, height, weight, BMI, fat mass, fat-free mass, waist-hip ratio, self-rated health, SMMSE, depressive symptoms, disease burden, total number of medications, and the intake of non-prescribed medication |
| Granic, 2016b | UK | The Newcastle 85+ Study | 768 | Old (85+ years) | F: 61.8%  M: 38.2% | 5 years | Average HGS (decline) | kg | Both hands | 5 | 1 | Sex, linear trend of time, dietary patterns, their interaction term, education, dominant hand, diet change in the past year, season-specific serum vit d quartiles, total energy, number of chronic diseases, BMI, physical activity, and smoking |
| Granic, 2017a | UK | The Newcastle 85+ Study | 813 | Old (85+ years) | F: 62.2% M: 37.8% | 5 years | Average HGS (decline) | kg | Both hands | 5 | 1 | Sex, height, BMI, fat-free mass, self-reported physical activity, self-rated health, presence of depressive symptoms, multi-morbidity, arthritis in hands, intake of non-steroidal anti-inflammatory drugs, attrition variable |
| Granic, 2017b | UK | The Newcastle 85+ Study | 754 | Old (85+ years) | F: 60% M: 40% | 5 years | Average HGS (decline) | kg | Both hands | 5 | 1 | Sex, height, BMI, fat-free mass, waist-to-hip ratio, self-rated health, number of chronic conditions, renal impairment, cognitive impairment, arthritis, use of walking aids, retention, physical activity, season of blood draw, vit D supplementation, taking vit D prescribed medication |
| Granic, 2018 | UK | The Newcastle 85+ Study | 722 | Old (85+ years) | F: 60%  M: 40% | 5 years | Average HGS (decline) | kg | Both hands | 5 | 2 | Sex, fat free mass, height, multimorbidity, self-rated health, cognitive impairment, arthritis in hands, PA, protein intake distribution, misreporting of food intake, attrition |
| Gray, 2012 | US | Women’s Health Initiative (WHI) | 5,777 | Old (65+ years) | F: 100% | 7.5 years (mean) | Average HGS (decline) | kg | Dominant hand | 4 | 1 | BMI, smoking, alcohol use, leisure time PA, diabetes, hypertension, coronary heart disease, depressive symptoms, physical function, other medications for hypertension, nonsteroidal anti-inflammatory drugs, menopausal hormone therapy |
| Gu, 2019 | China | Tianjin Chronic Low-grade Systemic Inflammation and Health (TCLSIH) Cohort Study | 2152 | Middle age (40-49 years) | F: 33.1%  M: 61.9% | 3 years (median) | Maximal and relative HGS (decline) | kg | Both hands | 4 | 1 | Age, sex, baseline BMI, smoking status, alcohol-drinking status, education levels, employment status, household income, physical activity, total energy intake, dietary patterns, depression symptom, and metabolic syndrome |
| Hengeveld, 2021 | Canada | Quebec NuAge Cohort | 1,098 | Old (67-84 years) | F: 52.3% M: 47.7% | 3 years | Maximal HGS (follow-up and decline) | kPa | Both hands | 2 | 1 | Age, height, weight, physical activity, education level, smoking, alcohol use, weight change, cognition, number of medications, and pain, and baseline HGS |
| Hilmer, 2009 | US | The Health, Aging, and Body Composition (Health ABC) Study | 2,099 | Old (70-79 years) | F: 52% M: 48% | 5 years | Maximal HGS (follow-up) | kg | Preferred hand | 2 | 1 | Age, sex, race, study site, education, comorbidities, hospitalisation, cognitive conditions, sleep problems, and baseline HGS |
| Hirani, 2016 | Australia | CHAMP | 1,666 | Old (70+ years) | M: 100% | 5 years | Maximal HGS (decline) | kg | Dominant hand | 3 | 1 | Age, income, BMI, comorbidities, cognitive status, depressive symptoms, cancer, estimated glomerular function, inflammatory markers (white blood cell and albumin), and medication use |
| Houston, 2012 | US | Health ABC Study | 2,522 | Old (70-79 years) | F: 51.1% M: 48.9% | 6 years | Maximal HGS (decline) | kg | Both hands | 3 | 1 | Age, sex, race, site, education, season; smoking status, alcohol intake, physical activity, BMI, kidney function, cognitive function, depressive symptoms, diabetes, CVD, COPD, knee pain, hospitalisation in the past year, multivitamin use, and vitamin D supplementation |
| Huang, 2021 | Japan | The Nagoya Longitudinal Study for Healthy Elderly | 666 | Old (60+ years) | F: 56.5% M: 43.5% | 3 years | Maximal HGS (decline) | kg | Both hands | 4 | 1 | Age, sex, BMI, educational level, economic status, CCI, BAQ, MNA, and total daily protein and energy intake |
| Hurst, 2021 | UK | UK Biobank study | 44,315 | Middle age (40-60 years) | F: 51.1% M: 48.9% | 9.2 years (median) | Maximal HGS (follow-up and decline) | kg | Both hands | 2 | 15 | HGS Z-scores based on age and sex |
| Ikeda, 2022 | Japan | ELSA | 5,080 | Middle age to old (50+ years) | F: 54.4% M: 45.6% | 4 years | Maximal HGS (follow-up) | kg | Dominant hand | 2 | 1 | Age, sex, ethnicity, educational attainment, marital status, equalized household income, sedentary behaviour, moderate PA, vigorous PA, longstanding illness, BMI, and baseline HGS |
| Jiang, 2022 | US | UK Biobank study | 42,843 | Middle age to old (40+ years) | F: 51% M: 49% | 9 years | Maximal HGS (follow-up) | kg | Dominant hand | 2 | 3 | Age, sex, education level, socioeconomic status, BMI, height, and waist-to-hip ratio |
| Kallman, 1990 | US | Baltimore Longitudinal Study of Aging (BLSA) | 847 | Young adult to old (20-100 years) | M: 100% | 9 years (mean) | Sum of maximal HGS (decline) | kg | Both hands | ≥3 | 2 | Age |
| Katzman, 2013 | US | The Study of Osteoporotic Fractures | 1,072 | Old (65+ years) | F: 100% | 15 years | Maximal HGS (decline) | kg | Dominant hand | 2 | 1 | Age, clinical site, weight, hypertension, arthritis, physical activity, alcohol, health status and baseline prevalent vertebral fracture |
| Keevil, 2015 | UK | The European Prospective Investigation of Cancer (EPIC)—Norfolk study | 5,569 | Middle age to old (48-92 years) | F: 55.6% M: 44.4% | 10 years | Maximal HGS (follow-up) | kg | Both hands | 1 | 1 | Age, sex, physical activity, anthropometry, wealth, comorbidity, smoking, and alcohol intake |
| Kim, 2017 | US | U.S. HRS | 4,486 | Middle age to old (50+ years) | F: 59.4% M: 40.6% | 4 years | Not described (follow-up and decline) | kg | Both hands | 2 | 1 | Age, sex, race/ethnicity, marital status, educational level, total wealth, baseline number of major chronic illnesses, depressive symptoms, and health behaviours (smoking, frequency of exercise, alcohol consumption). |
| Kim, 2019 | Korea | The Korean Longitudinal Study of Aging (KLoSA) | 5,995 | Middle age to old (50+ years) | F: 54.5% M: 45.5% | 8 years | Average HGS (follow-up) | kg | Both hands | 5 | 1 | Age, sex, education, total household income, education, alcohol consumption, smoking status, physical activity, obesity, co-morbidities, depressive symptoms, and engagement in social activities |
| Kim, 2021 | Korea | The Korean Genome and Epidemiology Study (KoGES) | 32,458 | Middle age (40-50 years) | F: 65% M: 35% | 4 years | Maximal HGS (follow-up) | kg | Both hands | 2 | 1 | Age, smoking status, alcohol consumption, physical activity, household income, educational level, BMI, total energy intake, histories of diabetes, hypertension, dyslipidaemia, cardio- and cerebrovascular diseases, or any cancer, laboratory tests, and lipid profiles |
| Kinoshita, 2022 | Japan | National Institute for Longevity Sciences-Longitudinal Study of Aging (NILS-LSA) | 701 | Old (60-83 years) | F: 46.5% M: 53.5% | 9.2 years | Maximal HGS (follow-up) | kg | Both hands | ≤5 | 1 | Sex, age, follow-up period, grip strength at baseline, BMI, PA, MMSE, education, smoking status, household income, hypertension, dyslipidaemia, diabetes, heart disease, PDCAAS for lunch and dinner, energy, protein intake at 33 regular meals |
| Koster, 2010 | US | Health ABC Study | 2,910 | Old (70-79 years) | F: 51.5% M: 48.5% | 8 years | Relative HGS (decline) | kg | Both hands | 8 | 1 | age, age2, race, site, and the interactions raceхage and siteхage |
| Kuh, 2002 | UK | MRC NSHD | 2,775 | Middle age (53 years) | F: 50.6% M: 49.4% | 53 years | Maximal HGS (follow-up) | kg | Both hands | 1 | 1 | Dynamometer/interviewer pair, current weight and height, weight and height at age 7y, and childhood and adulthood social class |
| Kuh, 2019 | UK | MRC NSHD | 3,058 | Middle age to old (53 and 69 years) | F: 50% M: 50% | 69 years | Maximal HGS (follow-up and decline) | kg | Both hands | 3 | 13 | Age, all growth parameters, adult height, BMI, health conditions, physical activity, smoking, adult socioeconomic position |
| Kurina, 2004 | US | The Study of Women’s Health Across the Nation (SWAN) | 563 | Middle age (42-52 years) | F: 100% | 3 years | Average HGS (decline) | kg | Both hands | 3 | 8 | Age. study visit, household income, BMI, smoking, and physical activity |
| Lauretani, 2008 | Italy | InCHIANTI Study | 948 | Old (65+ years) | F: 54.8% M: 45.2% | 6 years | Not described (follow-up) | kg | Both hands | 2 | 2 | Age, sex, education, BMI, WHR, calf muscle density, CSMA, current smoking, total energy intake, and physical activity |
| Lian, 2021 | China | China Health and Retirement Longitudinal Study (CHARLS) | 13,208 | Middle age to old (45+ years) | F: 53.7% M: 46.3% | 4 years | Maximal HGS (follow-up and decline) | kg | Both hands | 2-3 | 1 | Age, sex, education, marital status, smoking status, alcohol drinking status, BMI, sleep duration, number of chronic illnesses, and cognitive function |
| Luo, 2022 | China | U.S. HRS | 17,713 | Middle age to old (50+ years) | F: 56.2% M: 43.8% | 12 years | Maximal HGS (follow-up and decline) | kg | Both hands | 3 | 1 | Age, sex, current weakness situation, time, education levels, marital status, household income, physical activity level, drinking, and morbidity |
| Macchi, 2008 | Italy | InCHIANTI Study | 789 | Old (65+ years) | F: 54.5% M: 45.5% | 3 years | Not described (follow-up) | kg | Not described | 2 | 1 | Age, sex, BMI, hypertension, chronic heart failure, HDL cholesterol, triglycerides, creatinine, HOMA-R index, a- and g-tocopherol, C-reactive protein, interleukin 1 receptor antagonist, interleukin 6, vitamin C intake, number of medications, use of diuretics and baseline HGS |
| Mänty, 2014 | Denmark | Nationwide Danish 1905 cohort study | 1,353 | Old (92-93 years) | F: 72%  M: 28% | 5 years | Maximal HGS (decline) | kg | Preferred hand | 3 | 1 | Sex, housing, weight, and height |
| Mänty, 2015 | UK | MRC NSHD | 1,897 | Old (60-64 years) | F: 52.8% M: 47.2% | 17-21 years | Maximal HGS (follow-up) | kg | Both hands | 1 | 1 | sex, weight, height, occupational class, educational level, physical health, smoking, cumulative physical activity score |
| McLean, 2016 | US | The Framingham Offspring Cohort | 1,746 | Young adult to old (29-85 years) | F: 56.5% M: 43.5% | 5.8 years (mean) | Maximal HGS (decline) | kg | Both hands | 2 | 1 | Sex, age, height, total energy, BMI, physical activity, health status, and menopause status |
| Mejia, 2020 | US | U.S. HRS | 3,779 couples | Middle age to old (51+ years) | F: 50% M: 50% | 4 years | Average HGS (follow-up) | kg | Both hands | 2 | 1 | Partners’ age, race, and education, smoking, moderate physical activity, drinking, BMI, number of chronic conditions, number of depressive symptoms |
| Miller, 2010 | US | African American Health (AAH) project | 755 | Middle age (49-65 years) | F: 59% M: 41% | 3 years | Average HGS (decline) | kg | Preferred hand | 2 | 9 | Age, sex, marital status, education, income, perceived income adequacy, having Medicare, foregoing a |
| Møller, 2013 | Denmark | The Copenhagen Aging and Midlife Biobank (CAMB) | 3,843 | Middle age (53-64 years) | F: 20.8% M: 79.2% | Not described (retrospective cohort) | Maximal HGS (follow-up) | kg | Not described | 1 | 1 | Age, sex, Medicaid, unable to see doctor due to cost, CVD, ADL and IADL disabilities, lower body limitations, DBP, seasonally adjusted YPAS, BMI, and change in hand pain |
| Mulla, 2013 | UK | MRC NSHD | 1,806 | Middle age (53 years) | F: 51.4% M: 48.6% | 17 years | Maximal HGS (follow-up) | kg | Both hands | 1 | 4 | Sex, height, weight energy intake, childhood social class, adult social class, education and interaction terms between sex and height and sex and weight |
| Nahhas, 2010 | US | The Fels Longitudinal Study | 1,031 | Young adult to old (18-96 years) | F: 51.7% M: 48.3% | 23 years | Maximal HGS (follow-up and decline) | kg | Dominant hand | 1-11 | 5 | Age, maximum stature, and BMI at 45y |
| O'Keefe, 2022 | US | ELSA | 11,181 | Middle age to old (50+ years) | F: 55%  M: 45% | 12 years | Maximal HGS (decline) | kg | Dominant hand | 4 | 2 | Age, health, BMI, and physical activity |
| Okely, 2020 | UK | Lothian Birth Cohort 1936 | 1,091 | Old (70-79 years) | F: 49.8% M: 50.2% | 9 years | Maximal HGS (decline) | kg | Both hands | 4 | 1 | Age, sex, IQ at 11y, height, and history of diabetes, CVD, stroke, and hypertension |
| Oksuzyan, 2010 | Denmark | Danish 1905-cohort Study | 2,295 | Old (90+ years) | F: 74.5% M: 25.5% | 7 years | Maximal HGS (decline) | kg | Strongest hand | 4 | 1 | Not described - Age, sex |
| Paster, 2022 | US | Project Viva | 631 | Middle age (40-60 years) | F: 100% | 18.2 years (mean) | Average HGS (follow-up) | kg | Both hands | 1 | 1 | Age, race/ethnicity, education, marital status, ever smoking, household income at enrolment, and mother’s age at first pregnancy, diet, pre-pregnancy PA |
| Perala, 2017 | Finland | The Helsinki Birth Cohort Study | 1,072 | Old (70+ years) | F: 55.9% M: 44.1% | 10 years | Maximal HGS (follow-up) | N | Dominant hand | 1 | 5 | Age, energy intake, BMI, smoking status, educational attainment, and physical activity |
| Perri, 2020 | UK | The Newcastle 85+ Study | 791 | Old (85+ years) | F: 61.8% M: 38.1% | 5 years | Average HGS (decline) | kg | Both hands | 4 | 1 | Age at baseline, sex, occupational class, self-rated health, energy intake, protein intake, medication use, BMI, fat-free mass, physical activity, cognitive impairment, disability score, food intake misreports, and change in diet. |
| Proctor, 2006 | Sweden | OCTO-Twin Study | 579 | Old (79-96 years) | F: 67% M: 33% | 10 years | Maximal HGS (decline) | PSI | Both hands | 5 | 4 | Age, sex, and years to death |
| Raji, 2005 | US | The Hispanic Established Population for the Epidemiological Study of the Elderly (H-EPESE) | 2,381 | Old (65+ years) | F: 57% M: 43% | 7 years | Maximal HGS (follow-up and decline) | kg | Dominant hand | 4 | 8 | Age, sex, education, medical conditions, BMI, high depressive symptoms, and interaction terms (cognition by time and HGS over time) |
| Rantanen, 1998 | Hawaii | Honolulu Heart Program | 8,006 | Middle age to old (45-68 years) | M: 100% | 27 years | Maximal and average HGS (decline) | kg | Both hands | 4 | 8 | Age, height, and baseline HGS |
| Rantanen, 2000 | Hawaii | Honolulu-Asia Aging Study | 2,275 | Old (71-92 years) | M: 100% | 3 years | Maximal HGS (decline) | kg | Both hands | 2 | 2 | age, arthritis, angina, coronary heart disease, COPD, diabetes, stroke and exam 4 grip strength and weight, use of antidepressive medications |
| Reinders, 2015 | Iceland | The Age, Gene/Environment Susceptibility–Reykjavik Study | 836 | Old (66-96 years) | F: 54.3% M: 45.7% | 5 years | Maximal HGS (decline) | N | Both hands | 2 | 1 | Age, sex, physical activity, education, smoking status, BMI, diabetes, COPD, coronary heart disease, microalbuminuria, C-reactive protein, and time between assessments. |
| Ridgway, 2009 | Finland | The Northern Finland Birth Cohort 1966 (NFBC 1966) | 4,304 | Young adult (31 years) | F: 51.9% M: 48.1% | 31 years | Maximal HGS (follow-up) | kg | Dominant hand | 1 | 3 | sex, gestational age, father’s social class in 1966, and adult educational status, height, and weight |
| Ridgway, 2011 | Belgium | The East Flanders Prospective Twin Survey (EFPTS); the Prenatal Programming Twin Study | 783 | Young adult (18-34 years) | F: 51.2% M: 48.8% | 18-34 years | Maximal HGS (follow-up) | kg | Dominant hand | 1 | 1 | Sex, gestational age, adult age, adult height, adult fat free mass |
| Robinson, 2012 | UK | Hertfordshire Cohort Study (HCS) | 2983 | Middle age to old (59-73 years) | F: 47.4% M: 52.6% | 58-67 years | Maximal HGS (follow-up) | kg | Both hands | 1 | 3 | Age, sex, height, and physical activity score |
| Roh, 2022 | Korea | the Korean Frailty and Aging Cohort Study (KFACS) | 1,595 | Old (79-84 years) | F: 52.4% M: 47.6% | 2 years | Relative HGS (follow-up) | kg | Dominant hand | 2 | 1 | Age, sex, smoking, alcohol, physical activity, household income, hypertension, dyslipidaemia, chronic kidney disease, HOMA-IR, hs-CRP, and vitamin D levels |
| Sabia, 2014 | UK | the Whitehall II Study | 5,671 | Middle age to old (40+ years) | F: 27.9% M: 72.1% | 17 years | Maximal HGS (follow-up) | kg | Dominant hand | 1 | 4 | Age, sex, height, marital status, education, BMI at baseline |
| Sahni, 2021 | US | The Framingham Offspring Cohort | 2,452 | Young adult to old (33-88 years) | F: 55% M: 45% | 4.5-15.4 years | Maximal HGS (decline) | kg | Both hands | 2-3 | 1 | Age, sex, baseline HGS, height, BMI, energy, physical activity, current smoking, multivitamin use, and intake of other antioxidants |
| Sanders, 2010 | US | the Cardiovascular Health Study All Stars (CHS-All Stars) Study | 989 | Old (65+ years) | F: 63.5% M: 36.5% | 9 years | Not described (decline) | kg | Not described | 2 | 1 | Age, race, smoking, BMI, T-CT, number of medications, hypertension, kidney disease, COPD, cerebrovascular disease, coronary heart disease, diabetes, arthritis, cancer and depression, and baseline DHEAS |
| Sanders, 2014 | US | CHS-All Stars Study | 901 | Old (80+ years) | F: 64.9% M: 35.1% | 9 years | Average HGS (decline) | kg | Dominant hand | 2 | 2 | Age, sex, race, smoking status, marital status, number of chronic diseases, change in weight, and interaction of biomarker change with sex, if significant. |
| Santanasto, 2022 | Trinidad & Tobago | The Tobago Longitudinal Study of Aging | 1,918 | Middle age to old (40+ years) | M: 100% | 9 years | Maximal HGS (decline) | kg | Both hands | 2-3 | 2 | Age, height, weight, smoking status, and chronic diseases |
| Schaap, 2006 | Netherlands | The Longitudinal Aging Study Amsterdam (LASA) | 986 | Old (65+ years) | F: 52.7% M: 47.3% | 2 years | Average HGS (decline) | kg | Both hands | 2 | 1 | age and sex,r education level, smoking status, number of chronic diseases, alcohol use, physical activity, anti-inflammatory drug use, body mass index (or total body fat when available), cognitive impairment, and depressive symptoms. |
| Schalk, 2005 | Netherlands | LASA | 1,320 | Old (65+ years) | F: 51.2% M: 48.8% | 6 years | Maximal HGS (decline) | kg | Both hands | 3 | 1 | Age, smoking, alcohol, BMI, PA, diabetes, cardiac disease, stroke, arthritis, cognitive impairment, depression, total serum cholesterol, serum PTH, serum vit D, IL-6, CRP |
| Schneider, 2014 | Germany | SHARE | 20,929 | Middle age to old (50+ years) | F: 55.6% M: 44.4% | 1 year | Maximal HGS (follow-up) | kg | Both hands | 1 | 2 | Age, education, and country |
| Semba, 2012 | US | InCHIANTI study | 775 | Old (65+ years) | F: 55.8% M: 44.2% | 6 years | Not described (decline) | kg | both hands | 3 | 1 | Age, sex, education, smoking, physical activity, MMSE score, stroke, heart failure, chronic kidney disease, cancer, and depression |
| Shin, 2021 | South Korea | The Korean Frailty and Aging Cohort Study (KFACS) | 1,879 | Old (70-84 years) | F: 47.4% M: 52.6% | 2 years | Maximal HGS (decline) | kg | Both hands | 2 | 1 | Age, sex, waist circumference, smoking status, alcohol intake, number of comorbidities, physical activity, albumin, TGL, T-CT, HDL, haemoglobin, glycosylated haemoglobin, and log-transformed hs-CRP |
| Simpkin, 2017 | UK | MRC NSHD | 790 | Middle age (53 and 60-64 years) | F: 100% | 11 years | Maximal HGS (decline) | kg | Both hands | 2 | 1 | Age, height, BMI, smoking, and childhood and adult SEP |
| Skoog, 2016 | Sweden | The Prospective Population Study of Women (PPSW) and the Gerontological and Geriatric Population Studies (H70) | 607 | Old (75 and 79 years) | F: 59.8% M: 40.2% | 4 years | Maximal HGS (decline) | kg | Both hands | 2 | 1 | Sex, BMI at baseline, diabetes, T-CT at baseline, CVD, stroke, depression, MMSE score at baseline, change in MMSE score, and educational level |
| Smith, 2020 | UK | ELSA | 7,433 | Middle age to old (52+ years) | F: 53% M: 47% | 4 years | Maximal HGS (follow-up) | kg | Both hands | 2 | 1 | Age, sex, ethnicity, socioeconomic status, and BMI |
| Snyder, 2012 | US | Osteoporotic Fractures in Men (MrOS) Study | 1,267 | Old (65+ years) | M: 100% | 2 years (mean) | Average HGS (decline) | kg | Both hands | 3 | 1 | Age, study site, baseline ASM mass, and ASM mass change |
| Stenholm, 2010 | Italy | InCHIANTI study | 716 | Old (65+ years) | F: 56.7% M: 43.3% | 6 years | Maximal HGS (decline) | kg | Both hands | 3 | 3 | Age, sex, physical activity, and BMI |
| Stenholm, 2012 | Finland | Mini-Finland Health Examination Survey | 963 | Young adult to old (30-73 years) | F: 57.7%  M: 42.3% | 22.2 years (mean) | Maximal HGS (decline) | kg | Dominant hand | 2 | 18 | Age, sex, education, baseline BMI, physical activity, work-related physical activity, smoking, and alcohol use |
| Sternäng, 2015 | Sweden | Swedish Adoption/Twin Study of Aging (SATSA) | 849 | Middle age to old (50-88 years) | F: 59.3% M: 40.7% | 22 years | Maximal HGS (follow-up and decline) | kg | Both hands | ≤8 | 11 | Age, education level, SES, marital status, body weight, height, self-reported health, depression, stress, MAP, lipids, morbidity, smoking, and physical activity |
| Sternfeld, 2017 | US | SWAN | 1,769 | Middle age (42-52 years) | F: 100% | 16 years | Maximal HGS (follow-up) | kg (kilograms) | Dominant hand | 1 | 4 | Age, number of non-missing visits, race/ethnicity, BMI, overall self-rated health, and clinical site, dynamometer setting, education, marital status, difficulty paying for basics, and medical/health indicators |
| Strand, 2011 | UK | MRC NSHD | 2,318 | Middle age (53 years) | F: 52.1% M: 47.9% | 33 years | Not described (follow-up) | kg | Not described | 1 | 1 | Sex, height and weight at 53 y, education, adult SEP, lung function, health conditions, cognitive function at 53y |
| Strand, 2019 | Norway | Tromsø Study | 5,595 | Old (66-84 years) | F: 54% M: 46% | 9 years | Maximal HGS (follow-up) | bar | Non-dominant hand | 3 | 1 | Age, sex, height, weight, education, smoking, and physical activity |
| Swart, 2013 | Netherlands | LASA | 1,138 | Old (65+ years) | F: 51.5% M: 48.5% | 3 years | Average HGS (follow-up) | kg | both hands | 2 | 1 | gender, creatinine, BMI, region of living, education, alcohol consumption, smoking, serum vitamin B12 concentration |
| Syddall, 2018 | UK | ELSA; HCS | ELSA: 3,703 HCS: 441 | Middle age to old (50+ years) | F: 49.9-55.4%  M: 44.6-50.1% | ELSA: 6 years HCS: 10.6 years | Maximal HGS (decline) | kg | Both hands | 2-3 | 13 | Age, sex, height, and weight-for-height residual |
| Taeymans, 2009 | Switzerland | Longitudinal Experimental Growth Study (LEGS) | 119 | Young adult (35 years) | F: 50.4% M: 49.6% | 29 years | Maximal HGS (follow-up) | kg | Not described | ≤14 | 1 | Sex |
| Tiainen, 2022 | Finland | Finland Health 2000 Survey | 1,200 | Middle age to old (55–86 years) | F: 56.6% M: 43.4% | 11 years | Maximal HGS (decline) | N | Dominant hand | 2 | 1 | Age, sex, education, baseline BMI, HRT, physical activity, alcohol consumption, and smoking status, baseline chronic conditions, follow-up diabetes status, baseline HGS |
| van Ballegooijen, 2018 | Netherlands | LASA | 633 | Middle age (55-65 years) | F: 54% M: 46% | 11.1 years (mean) | Average HGS (follow-up) | kg | Both hands | ≥2 | 1 | Baseline age, sex, follow-up time, baseline number of chronic diseases, BMI, education, alcohol consumption, smoking status, physical activity, depression, type 2 diabetes, hypertension, vit D and estimate glomerular filtration rate |
| van Nieuwpoort, 2018 | Netherlands | LASA | 1,292 | Old (65+ years) | F: 51%  M: 49% | 3 years | Average HGS (decline) | kg | Both hands | 2 | 1 | BMI, age, smoking status, alcohol intake, chronic diseases, albumin, creatinine, testosterone, oestradiol, level of education and physical activity score. Stratified by gender and physical activity score. |
| Veronese, 2017 | Italy | Progetto Veneto Anziani (PRO.V.A.) study | 1,904 | Old (65+ years) | F: 62.5% M: 37.5% | 4.4 years (mean) | Maximal HGS (follow-up) | kg | Both hands | 2 | 1 | Age, BMI, eGFR, number of ADLs preserved, GDS and MMSE scores, diabetes, hypertension, CVDs, fractures, osteoarthritis, COPD, and cancer at baseline, educational level, smoking habits, monthly income, alcohol drinking, self-reported physical activity level, and baseline value of HGS |
| Vidoni, 2018 | US | BLSA | 1,101 | Middle age to old (50+ years) | F: 49.5% M: 50.5% | 4.7 years (mean) | Maximal HGS (decline) | kg | Both hands | ≥1 | 1 | follow-up time, baseline age category, race, physical activity, and BMI categories |
| Visser, 2003 | Netherlands | LASA | 1,008 | Old (65+ years) | F: 52.6% M: 47.3% | 3 years | Sum of maximal HGS (decline) | kg | Both hands | 2 | 2 | Age, sex, height, BMI, physical activity level, serum creatinine concentration, season of data collection, chronic disease, and smoking status |
| Wang, 2018 | China | CHARLS | 9,019 | Middle age to old (45+ years) | F: 53% M: 47% | 4 years (mean) | Sum of maximal HGS (decline) | kg | Both hands | 2 | 1 | Age, educational level, alcohol drinking, cigarette smoking, BMI, mental disorder, stroke, hypertension, type 2 diabetes, and cardiovascular disease |
| Wang, 2019 | China | SHARE | 18,704 | Middle age to old (50+ years) | F: 53.9% M: 46.1% | 2 years | Maximal HGS (decline) | kg | Both hands | 5 | 2 | Age, country, educational level, cigarette smoking, alcohol drinking, depression, heart attack, arthritis, hypertension, hypercholesterolaemia, and diabetes |
| Witham, 2014 | UK | HCS | 639 | Old (60+ years) | F: 49.7% M: 50.2% | 4.4 years (mean) | Maximal HGS (decline) | kg | Both hands | 2 | 1 | Age, sex, height, weight, baseline grip strength, indices of ischaemic heart disease and hypertension |
| Woo, 2014 | China | Cohort from China | 2,006 | Old (65+ years) | F: 51.3% M: 48.7% | 5 years | Maximal HGS (decline) | kg | Both hands | 2 | 1 | age, education level, BMI, smoking, physical activity, and probable dementia |
| Ylihärsilä, 2007 | Finland | birth cohort from Finland | 2,003 | Middle age to old (55+ years) | F: 53.7% M: 46.3% | 56-66 years | Maximal HGS (follow-up) | kg | Dominant hand | 1 | 1 | Age and height or age and adult BMI |
| Yokoyama, 2017 | Japan | Hatoyama Cohort Study and Kusatsu Longitudinal Study | 779 | Old (65+ years) | F: 46.6% M: 53.4% | 4 years | Maximal HGS (decline) | kg | Dominant hand | 2 | 1 | Age, sex, education, living status, self-perceived chewing ability, smoking habit, drinking habit, exercise habit, BMI, depressive symptoms, cognitive status, physician-diagnosed illnesses |
| Yun, 2022 | Korea | KLoSA | 3,843 | Middle age to old (45+ years) | F: 45.2% M: 54.8% | 12 years | Average HGS (follow-up) | kg | Both hands | 7 | 1 | Age, sex, marital status, region, education, income, self-reported health, BMI, ADL, IADL, cognitive function, physical activity |
| Yusuf, 2022 | UK | 1970 British Cohort Study (BCS70) | 7,617 | Middle age (46 years) | F: 51.5% M: 48.5% | 3 years | Maximal HGS (follow-up) | kg | Both hands | 1 | 1 | Birth weight, BMI in childhood and adulthood, adult height, disability in childhood, leisure-time physical activity in childhood and adulthood, sedentary behaviour in childhood and adulthood, occupational activity, and smoking at 46y |
| Zhang, 2021 | China | CHARLS | 2,418 | Old (60+ years) | F: 49.1% M: 50.9% | 4 years | Maximal HGS (follow-up) | kg | Both hands | 2 | 4 | Age, sex, marital status, urban residence, education, medical insurance, per capita expenditure (PCE), chronic lung disease, cancer, heart problems, stroke, arthritis, memory-related diseases, depression, smoking, drinking, and regular physical exercise |
| Zhang, 2022 | China | TCLSIH | 5,409 | Middle age to old (40+ years) | F: 38.7% M: 61.3% | At least 1 year | Maximal and relative HGS (decline) | kg (kilograms) | Both hands | ≥2 | 1 | Age, sex, and BMI, smoking status, alcohol drinking status, education level, employment, monthly household income, physical activity, family history of disease, depressive symptoms, hypertension, hyperlipidaemia, diabetes, total energy intake, dietary supplement usage, total protein intake, milk intake, and healthy diet score, baseline grip strength or baseline weight-adjusted grip strength |

F: female; M: male; N: Newtons; kg: kilograms; kgf: kilogram force; kg/cm^2^: kilogram force per square centimetre; kPa: kilopascal; PSI: pound per square inch.

# References

1. Beller J, Miething A, Regidor E, Lostao L, Epping J, Geyer S. Trends in grip strength: Age, period, and cohort effects on grip strength in older adults from Germany, Sweden, and Spain. SSM - population health. 2019;9:100456.

2. Forrest KYZ, Zmuda JM, Cauley JA. Patterns and correlates of muscle strength loss in older women. Gerontology. 2007;53(3):140-7.

3. Forrest KYZ, Bunker CH, Sheu Y, Wheeler VW, Patrick AL, Zmuda JM. Patterns and correlates of grip strength change with age in Afro-Caribbean men. Age and Ageing. 2012;41(3):326-32.

4. Kallman DA, Plato CC, Tobin JD. The role of muscle loss in the age-related decline of grip strength: Cross-sectional and longitudinal perspectives. Journals of Gerontology. 1990;45(3):M82-M8.

5. Kurina LM, Gulati M, Everson-Rose SA, Chung PJ, Karavolos K, Cohen NJ, et al. The effect of menopause on grip and pinch strength: results from the Chicago, Illinois, site of the Study of Women's Health Across the Nation. American journal of epidemiology. 2004;160(5):484-91.

6. Miller DK, Malmstrom TK, Miller JP, Andresen EM, Schootman M, Wolinsky FD. Predictors of change in grip strength over 3 years in the African American health project. J Aging Health. 2010;22(2):183-96.

7. O'Keefe P, Mann FD, Clouston S, Voll S, Muniz-Terrera G, Lewis N, et al. Getting a Grip on Secular Changes: Age-Period-Cohort Modeling of Grip Strength in the English Longitudinal Study of Ageing. The journals of gerontology Series A, Biological sciences and medical sciences. 2022;77(7):1413-20.

8. Proctor DN, Fauth EB, Hoffman L, Hofer SM, McClearn GE, Berg S, et al. Longitudinal changes in physical functional performance among the oldest old: insight from a study of Swedish twins. Aging clinical and experimental research. 2006;18(6):517-30.

9. Raji MA, Kuo Y-F, Snih SA, Markides KS, Peek MK, Ottenbacher KJ. Cognitive status, muscle strength, and subsequent disability in older Mexican Americans. Journal of the American Geriatrics Society. 2005;53(9):1462-8.

10. Rantanen T, Masaki K, Foley D, Izmirlian G, White L, Guralnik JM. Grip strength changes over 27 yr in Japanese-American men. Journal of Applied Physiology. 1998;85(6):2047-53.

11. Stenholm S, Tiainen K, Rantanen T, Sainio P, Heliövaara M, Impivaara O, et al. Long-term determinants of muscle strength decline: Prospective evidence from the 22-year Mini-Finland follow-up survey. Journal of the American Geriatrics Society. 2012;60(1):77-85.

12. Sternäng O, Reynolds CA, Finkel D, Ernsth-bravell M, Pedersen NL, Dahl aslan AK. Factors associated with grip strength decline in older adults. Age and Ageing. 2015;44(2):269-74.

13. Syddall HE, Westbury LD, Shaw SC, Dennison EM, Cooper C, Gale CR. Correlates of Level and Loss of Grip Strength in Later Life: Findings from the English Longitudinal Study of Ageing and the Hertfordshire Cohort Study. Calcified tissue international. 2018;102(1):53-63.

14. Granic A, Davies K, Jagger C, Kirkwood TBL, Syddall HE, Sayer AA. Grip Strength Decline and Its Determinants in the Very Old: Longitudinal Findings from the Newcastle 85+ Study. PloS one. 2016;11(9):e0163183.

15. Oksuzyan A, Maier H, McGue M, Vaupel JW, Christensen K. Sex differences in the level and rate of change of physical function and grip strength in the Danish 1905-cohort study. Journal of aging and health. 2010;22(5):589-610.

16. Schneider B, Rapp I, Klein T, Eckhard J. Relationship status and health: Does the use of different relationship indicators matter? Global public health. 2014;9(5):528-37.

17. Nahhas RW, Choh AC, Lee M, Chumlea WM, Duren DL, Siervogel RM, et al. Bayesian longitudinal plateau model of adult grip strength. Am J Hum Biol. 2010;22(5):648-56.

18. Strand BH, Bergland A, Jørgensen L, Schirmer H, Emaus N, Cooper R. Do More Recent Born Generations of Older Adults Have Stronger Grip? A Comparison of Three Cohorts of 66- to 84-Year-Olds in the Tromsø Study. The journals of gerontology Series A, Biological sciences and medical sciences. 2019;74(4):528-33.

19. Botoseneanu A, Bennett JM, Nyquist L, Shinkai S, Fujiwara Y, Yoshida H, et al. Cardiometabolic Risk, Socio-Psychological Factors, and Trajectory of Grip Strength Among Older Japanese Adults. Journal of aging and health. 2015;27(7):1123-46.

20. Kuh D, Hardy R, Blodgett JM, Cooper R. Developmental factors associated with decline in grip strength from midlife to old age: A British birth cohort study. BMJ Open. 2019;9(5).

21. Yun I, Park YS, Park E-C, Jang S-I. Association between changes in working status and hand-grip strength among Korean middle-aged and older adults: a longitudinal panel study. Scientific reports. 2022;12(1):12897.

22. de Vries R, Blane D, Netuveli G. Long-term exposure to income inequality: implications for physical functioning at older ages. European journal of ageing. 2013;11(1):19-29.

23. Chang X, Chua KY, Wang L, Liu J, Yuan J-M, Khor C-C, et al. Midlife Leukocyte Telomere Length as an Indicator for Handgrip Strength in Late Life. The journals of gerontology Series A, Biological sciences and medical sciences. 2021;76(1):172-5.

24. Semba R, Cappola A, Sun K, Bandinelli S, Dalal M, Crasto C, et al. Relationship of low plasma klotho with poor grip strength in older community-dwelling adults: the InCHIANTI study. European Journal of Applied Physiology. 2012;112(4):1215-20.

25. Simpkin AJ, Cooper R, Howe LD, Relton CL, Davey Smith G, Teschendorff A, et al. Are objective measures of physical capability related to accelerated epigenetic age? Findings from a British birth cohort. BMJ open. 2017;7(10):e016708.

26. Woo J, Yu R, Tang N, Leung J. Telomere length is associated with decline in grip strength in older persons aged 65 years and over. Age (Dordrecht, Netherlands). 2014;36(5):9711.

27. Batterham PJ, Bunce D, Cherbuin N, Christensen H. Apolipoprotein E ε4 and later-life decline in cognitive function and grip strength. The American journal of geriatric psychiatry : official journal of the American Association for Geriatric Psychiatry. 2013;21(10):1010-9.

28. Skoog I, Hörder H, Frändin K, Johansson L, östling S, Blennow K, et al. Association between APOE genotype and change in physical function in a population-based swedish cohort of older individuals followed over four years. Frontiers in Aging Neuroscience. 2016;8(OCT).

29. Ahlqvist VH, Persson M, Ortega FB, Tynelius P, Magnusson C, Berglind D. Birth weight and grip strength in young Swedish males: a longitudinal matched sibling analysis and across all body mass index ranges. Scientific reports. 2019;9(1):9719.

30. Bielemann RM, Gigante DP, Horta BL. Birth weight, intrauterine growth restriction and nutritional status in childhood in relation to grip strength in adults: From the 1982 Pelotas (Brazil) birth cohort. Nutrition. 2016;32(2):228-35.

31. Kuh D, Bassey J, Hardy R, Aihie Sayer A, Wadsworth M, Cooper C. Birth weight, childhood size, and muscle strength in adult life: evidence from a birth cohort study. American journal of epidemiology. 2002;156(7):627-33.

32. Ridgway CL, Ong KK, Tammelin T, Sharp SJ, Ekelund U, Jarvelin M-R. Birth size, infant weight gain, and motor development influence adult physical performance. Medicine and science in sports and exercise. 2009;41(6):1212-21.

33. Ridgway CL, Sharp SJ, Derom C, Beunen G, Fagard R, Vlietinck R, et al. The contribution of prenatal environment and genetic factors to the association between birth weight and adult grip strength. PloS one. 2011;6(3):e17955.

34. Robinson SM, Simmonds SJ, Jameson KA, Syddall HE, Dennison EM, Cooper C, et al. Muscle strength in older community-dwelling men is related to type of milk feeding in infancy. The journals of gerontology Series A, Biological sciences and medical sciences. 2012;67(9):990-6.

35. Ylihärsilä H, Kajantie E, Osmond C, Forsén T, Barker DJP, Eriksson JG. Birth size, adult body composition and muscle strength in later life. International journal of obesity (2005). 2007;31(9):1392-9.

36. Du T, Fernandez C, Barshop R, Guralnik J, Bazzano LA. Cardiovascular risk factors from childhood and midlife physical function: The Bogalusa Heart Study. Experimental gerontology. 2020;136:110947.

37. Yusuf M, Montgomery G, Hamer M, McPhee J, Cooper R. Associations between childhood and adulthood socioeconomic position and grip strength at age 46 years: findings from the 1970 British Cohort Study. BMC public health. 2022;22(1):1427.

38. Cooper R, Richards M, Kuh D. Childhood Cognitive Ability and Age-Related Changes in Physical Capability From Midlife: Findings From a British Birth Cohort Study. Psychosom Med. 2017;79(7):785-91.

39. Cheval B, Chabert C, Sieber S, Orsholits D, Cooper R, Guessous I, et al. Association between Adverse Childhood Experiences and Muscle Strength in Older Age. Gerontology. 2019;65(5):474-84.

40. de Carvalho DHT, Scholes S, Santos JLF, de Oliveira C, Alexandre TdS. Does Abdominal Obesity Accelerate Muscle Strength Decline in Older Adults? Evidence From the English Longitudinal Study of Ageing. The journals of gerontology Series A, Biological sciences and medical sciences. 2019;74(7):1105-11.

41. Hurst C, Murray JC, Granic A, Hillman SJ, Cooper R, Sayer AA, et al. Long-term conditions, multimorbidity, lifestyle factors and change in grip strength over 9 years of follow-up: Findings from 44,315 UK biobank participants. Age and Ageing. 2021;50(6):2222-9.

42. Rantanen T, Penninx BW, Masaki K, Lintunen T, Foley D, Guralnik JM. Depressed mood and body mass index as predictors of muscle strength decline in old men. Journal of the American Geriatrics Society. 2000;48(6):613-7.

43. Wang T, Feng W, Li S, Tan Q, Zhang D, Wu Y. Impact of obesity and physical inactivity on the long-term change in grip strength among middle-aged and older European adults. Journal of epidemiology and community health. 2019;73(7):619-24.

44. Santanasto AJ, Miljkovic I, Cvejkus RK, Boudreau RM, Wheeler VW, Zmuda JM. Body Composition Across the Adult Lifespan in African Caribbean Men: The Tobago Longitudinal Study of Aging. The Journal of frailty & aging. 2022;11(1):40-4.

45. Zhang Q, Zhao X, Liu H, Yu N, Li D. Association between the metabolic syndrome and muscle weakness among Chinese older adults: results from the China Health and Retirement Longitudinal Study. Geriatric nursing (New York, NY). 2021;42(6):1415-21.

46. Cooper R, Hardy R, Bann D, Aihie Sayer A, Ward KA, Adams JE, et al. Body mass index from age 15 years onwards and muscle mass, strength, and quality in early old age: findings from the MRC National Survey of Health and Development. The journals of gerontology Series A, Biological sciences and medical sciences. 2014;69(10):1253-9.

47. Granic A, Davies K, Martin-Ruiz C, Jagger C, Kirkwood TBL, von Zglinicki T, et al. Grip strength and inflammatory biomarker profiles in very old adults. Age and ageing. 2017;46(6):976-82.

48. Hirani V, Naganathan V, Blyth F, Le Couteur DG, Seibel MJ, Waite LM, et al. Low Hemoglobin Concentrations Are Associated With Sarcopenia, Physical Performance, and Disability in Older Australian Men in Cross-sectional and Longitudinal Analysis: The Concord Health and Ageing in Men Project. The journals of gerontology Series A, Biological sciences and medical sciences. 2016;71(12):1667-75.

49. Sanders JL, Ding V, Arnold AM, Kaplan RC, Cappola AR, Kizer JR, et al. Do changes in circulating biomarkers track with each other and with functional changes in older adults? The journals of gerontology Series A, Biological sciences and medical sciences. 2014;69(2):174-81.

50. Schaap LA, Pluijm SMF, Deeg DJH, Visser M. Inflammatory Markers and Loss of Muscle Mass (Sarcopenia) and Strength. American Journal of Medicine. 2006;119(6):526.e9-.e17.

51. Schalk BWM, Deeg DJH, Penninx BWJH, Bouter LM, Visser M. Serum albumin and muscle strength: A longitudinal study in older men and women. Journal of the American Geriatrics Society. 2005;53(8):1331-8.

52. Snyder CK, Lapidus JA, Cawthon PM, Dam T-TL, Sakai LY, Marshall LM. Serum albumin in relation to change in muscle mass, muscle strength, and muscle power in older men. Journal of the American Geriatrics Society. 2012;60(9):1663-72.

53. Stenholm S, Maggio M, Lauretani F, Bandinelli S, Ceda GP, Di Iorio A, et al. Anabolic and catabolic biomarkers as predictors of muscle strength decline: The InCHIANTI study. Rejuvenation Research. 2010;13(1):3-11.

54. Swart KMA, Van Schoor NM, Heymans MW, Schaap LA, Den Heijer M, Lips P. Elevated homocysteine levels are associated with low muscle strength and functional limitations in older persons. The Journal of nutrition, health & aging. 2013;17(6):578-84.

55. Vidoni ML, Pettee Gabriel K, Luo ST, Simonsick EM, Day RS. Relationship between Homocysteine and Muscle Strength Decline: The Baltimore Longitudinal Study of Aging. The journals of gerontology Series A, Biological sciences and medical sciences. 2018;73(4):546-51.

56. Macchi C, Molino-Lova R, Polcaro P, Guarducci L, Lauretani F, Cecchi F, et al. Higher circulating levels of uric acid are prospectively associated with better muscle function in older persons. Mechanisms of Ageing and Development. 2008;129(9):522-7.

57. Veronese N, Stubbs B, Trevisan C, Bolzetta F, De Rui M, Maggi S, et al. Results of an Observational Cohort Study of Hyperuricemia as a Predictor of Poor Physical Performance in the Elderly. Arthritis care & research. 2017;69(8):1238-44.

58. García-Esquinas E, Carrasco-Rios M, Ortolá R, Sotos Prieto M, Pérez-Gómez B, Gutiérrez-González E, et al. Selenium and impaired physical function in US and Spanish older adults. Redox Biology. 2021;38.

59. Lauretani F, Semba RD, Bandinelli S, Dayhoff-Brannigan M, Giacomini V, Corsi AM, et al. Low plasma carotenoids and skeletal muscle strength decline over 6 years. Journals of Gerontology - Series A Biological Sciences and Medical Sciences. 2008;63(4):376-83.

60. Reinders I, Song X, Visser M, Eiriksdottir G, Gudnason V, Sigurdsson S, et al. Plasma phospholipid PUFAs are associated with greater muscle and knee extension strength but not with changes in muscle parameters in older adults. Journal of Nutrition. 2015;145(1):105-12.

61. Sanders JL, Cappola AR, Arnold AM, Boudreau RM, Chaves PH, Robbins J, et al. Concurrent change in dehydroepiandrosterone sulfate and functional performance in the oldest old: results from the Cardiovascular Health Study All Stars study. The journals of gerontology Series A, Biological sciences and medical sciences. 2010;65(9):976-81.

62. van Nieuwpoort IC, Vlot MC, Schaap LA, Lips P, Drent ML. The relationship between serum IGF-1, handgrip strength, physical performance and falls in elderly men and women. European journal of endocrinology. 2018;179(2):73-84.

63. Granic A, Hil TR, Davies K, Jagger C, Adamson A, Siervo M, et al. Vitamin d status, muscle strength and physical performance decline in very old adults: A prospective study. Nutrients. 2017;9(4).

64. Houston DK, Tooze JA, Neiberg RH, Hausman DB, Johnson MA, Cauley JA, et al. 25-hydroxyvitamin D status and change in physical performance and strength in older adults: the Health, Aging, and Body Composition Study. American journal of epidemiology. 2012;176(11):1025-34.

65. Visser M, Deeg DJH, Lips P. Low Vitamin D and High Parathyroid Hormone Levels as Determinants of Loss of Muscle Strength and Muscle Mass (Sarcopenia): The Longitudinal Aging Study Amsterdam. Journal of Clinical Endocrinology and Metabolism. 2003;88(12):5766-72.

66. Koster A, Visser M, Simonsick EM, Yu B, Allison DB, Newman AB, et al. Association between fitness and changes in body composition and muscle strength. Journal of the American Geriatrics Society. 2010;58(2):219-26.

67. Gu Y, Meng G, Wu H, Zhang Q, Liu L, Bao X, et al. Thyroid Function as a Predictor of Handgrip Strength Among Middle-Aged and Older Euthyroid Adults: The TCLSIH Cohort Study. Journal of the American Medical Directors Association. 2019;20(10):1236-41.

68. van Ballegooijen AJ, van Putten SR, Visser M, Beulens JW, Hoogendijk EO. Vitamin K status and physical decline in older adults-The Longitudinal Aging Study Amsterdam. Maturitas. 2018;113:73-9.

69. Cooper R, Muniz-Terrera G, Kuh D. Associations of behavioural risk factors and health status with changes in physical capability over 10 years of follow-up: the MRC National Survey of Health and Development. BMJ Open. 2016;6(4):e009962.

70. Ferrari U, Then C, Rottenkolber M, Selte C, Seissler J, Conzade R, et al. Longitudinal association of type 2 diabetes and insulin therapy with muscle parameters in the KORA-Age study. Acta diabetologica. 2020;57(9):1057-63.

71. Tiainen K, Raitanen J, Strandberg T, Koskinen S, Stenholm S. Type 2 Diabetes as a Predictor of Muscle Strength Decline over 11 years among Men and Women Aged 55 Years and Older. Gerontology. 2022;68(6):635-43.

72. Gray SL, Aragaki AK, Lamonte MJ, Cochrane BB, Kooperberg C, Robinson JG, et al. Statins, angiotensin-converting enzyme inhibitors, and physical performance in older women. Journal of the American Geriatrics Society. 2012;60(12):2206-14.

73. Hilmer SN, Mager DE, Simonsick EM, Ling SM, Windham BG, Harris TB, et al. Drug Burden Index Score and Functional Decline in Older People. American Journal of Medicine. 2009;122(12):1142-9.e2.

74. Witham MD, Syddall HE, Dennison E, Cooper C, McMurdo MET, Sayer AA. ACE inhibitors, statins and thiazides: no association with change in grip strength among community dwelling older men and women from the Hertfordshire Cohort Study. Age and ageing. 2014;43(5):661-6.

75. Katzman WB, Huang M-H, Lane NE, Ensrud KE, Kado DM. Kyphosis and decline in physical function over 15 years in older community-dwelling women: the Study of Osteoporotic Fractures. The journals of gerontology Series A, Biological sciences and medical sciences. 2013;68(8):976-83.

76. Smith L, Allen P, Pardhan S, Gorely T, Grabovac I, Smith A, et al. Self-rated eyesight and handgrip strength in older adults. Wiener klinische Wochenschrift. 2020;132(5-6):132-8.

77. Roh E, Hwang SY, Yoo HJ, Baik SH, Lee JH, Son SJ, et al. Impact of non-alcoholic fatty liver disease on the risk of sarcopenia: a nationwide multicenter prospective study. Hepatology International. 2022;16(3):545-54.

78. Mänty M, Ekmann A, Thinggaard M, Christensen K, Avlund K. Indoor mobility-related fatigue and muscle strength in nonagenarians: A prospective longitudinal study. Aging Clinical and Experimental Research. 2014;26(1):39-46.

79. Mänty M, Kuh D, Cooper R. Associations of Midlife to Late Life Fatigue with Physical Performance and Strength in Early Old Age: Results from a British Prospective Cohort Study. Psychosomatic Medicine. 2015;77(7):823-32.

80. Bertoni M, Maggi S, Manzato E, Veronese N, Weber G. Depressive symptoms and muscle weakness: A two-way relation? Experimental gerontology. 2018;108:87-91.

81. Ikeda T, Tsuboya T. Effects of changes in depressive symptoms on handgrip strength in later life: A four-year longitudinal study in England. Journal of affective disorders. 2022;299:67-72.

82. Jiang R, Westwater ML, Noble S, Rosenblatt M, Dai W, Qi S, et al. Associations between grip strength, brain structure, and mental health in > 40,000 participants from the UK Biobank. BMC Medicine. 2022;20(1).

83. Lian Y, Wang G-P, Chen G-Q, Jia C-X. Bidirectional Associations between Handgrip Strength and Depressive Symptoms: A Longitudinal Cohort Study. Journal of the American Medical Directors Association. 2021;22(8):1744.

84. Luo J, Yao W, Zhang T, Ge H, Zhang D. Exploring the bidirectional associations between handgrip strength and depression in middle and older Americans. Journal of Psychosomatic Research. 2022;152.

85. Kim GR, Sun J, Han M, Nam CM, Park S. Evaluation of the directional relationship between handgrip strength and cognitive function: the Korean Longitudinal Study of Ageing. Age and ageing. 2019;48(3):426-32.

86. Okely JA, Deary IJ. Associations Between Declining Physical and Cognitive Functions in the Lothian Birth Cohort 1936. The journals of gerontology Series A, Biological sciences and medical sciences. 2020;75(7):1393-402.

87. Kim ES, Kawachi I, Chen Y, Kubzansky LD. Association between purpose in life and objective measures of physical function in older adults. JAMA Psychiatry. 2017;74(10):1039-45.

88. Mejía ST, Giasson HL, Smith J, Gonzalez R. Concurrent and enduring associations between married partners’ shared beliefs and markers of aging. Psychology and Aging. 2020;35(7):925-36.

89. Cooper A, Lamb M, Sharp SJ, Simmons RK, Griffin SJ. Bidirectional association between physical activity and muscular strength in older adults: Results from the UK Biobank study. International journal of epidemiology. 2017;46(1):141-8.

90. Cooper R, Mishra GD, Kuh D. Physical activity across adulthood and physical performance in midlife: findings from a British birth cohort. American journal of preventive medicine. 2011;41(4):376-84.

91. de Lima TR, González-Chica DA, D'Orsi E, Sui X, Silva DAS. Individual and Combined Association Between Healthy Lifestyle Habits With Muscle Strength According to Cardiovascular Health Status in Adults and Older Adults. Journal of physical activity & health. 2021;18(8):973-80.

92. Granic A, Mendonça N, Sayer AA, Hill TR, Davies K, Adamson A, et al. Low protein intake, muscle strength and physical performance in the very old: The Newcastle 85+ Study. Clinical Nutrition. 2018;37(6):2260-70.

93. Sternfeld B, Colvin A, Stewart A, Dugan S, Nackers L, El Khoudary SR, et al. The Effect of a Healthy Lifestyle on Future Physical Functioning in Midlife Women. Medicine & Science in Sports & Exercise. 2017;49(2):274-82.

94. Sabia S, Elbaz A, Rouveau N, Brunner EJ, Kivimaki M, Singh-Manoux A. Cumulative associations between midlife health behaviors and physical functioning in early old age: a 17-year prospective cohort study. Journal of the American Geriatrics Society. 2014;62(10):1860-8.

95. Strand BH, Mishra G, Kuh D, Guralnik JM, Patel KV. Smoking history and physical performance in midlife: results from the British 1946 birth cohort. The journals of gerontology Series A, Biological sciences and medical sciences. 2011;66(1):142-9.

96. Perälä M-M, von Bonsdorff MB, Männistö S, Salonen MK, Simonen M, Kanerva N, et al. The healthy Nordic diet predicts muscle strength 10 years later in old women, but not old men. Age and ageing. 2017;46(4):588-94.

97. Hengeveld LM, Chevalier S, Visser M, Gaudreau P, Presse N. Prospective associations of protein intake parameters with muscle strength and physical performance in community-dwelling older men and women from the Quebec NuAge cohort. American Journal of Clinical Nutrition. 2021;113(4):972-83.

98. Kinoshita K, Otsuka R, Nishita Y, Tange C, Tomida M, Zhang S, et al. Breakfast Protein Quality and Muscle Strength in Japanese Older Adults: A Community-Based Longitudinal Study. Journal of the American Medical Directors Association. 2022;23(5):729-35.e2.

99. Kim H-N, Kim S-H, Eun Y-M, Song S-W. Impact of dietary protein intake on the incidence of low muscle strength in middle-aged and older adults. Clinical nutrition (Edinburgh, Scotland). 2021;40(4):1467-74.

100. McLean RR, Mangano KM, Hannan MT, Kiel DP, Sahni S. Dietary Protein Intake Is Protective Against Loss of Grip Strength Among Older Adults in the Framingham Offspring Cohort. The journals of gerontology Series A, Biological sciences and medical sciences. 2016;71(3):356-61.

101. Mulla UZ, Cooper R, Mishra GD, Kuh D, Stephen AM. Adult macronutrient intake and physical capability in the MRC National Survey of Health and Development. Age and ageing. 2013;42(1):81-7.

102. Keevil VL, Wijndaele K, Luben R, Sayer AA, Wareham NJ, Khaw K-T. Television viewing, walking speed, and grip strength in a prospective cohort study. Medicine and science in sports and exercise. 2015;47(4):735-42.

103. Dodds R, Kuh D, Aihie Sayer A, Cooper R. Physical activity levels across adult life and grip strength in early old age: updating findings from a British birth cohort. Age and ageing. 2013;42(6):794-8.

104. Møller A, Reventlow S, Hansen ÅM, Andersen LL, Siersma V, Lund R, et al. Does a history of physical exposures at work affect hand-grip strength in midlife? A retrospective cohort study in Denmark. Scandinavian journal of work, environment & health. 2013;39(6):599-608.

105. Cervo MMC, Scott D, Seibel MJ, Cumming RG, Naganathan V, Blyth FM, et al. Adherence to Mediterranean diet and its associations with circulating cytokines, musculoskeletal health and incident falls in community-dwelling older men: The Concord Health and Ageing in Men Project. Clinical nutrition (Edinburgh, Scotland). 2021;40(12):5753-63.

106. Huang CH, Okada K, Matsushita E, Uno C, Satake S, Arakawa Martins B, et al. Dietary Patterns and Muscle Mass, Muscle Strength, and Physical Performance in the Elderly: A 3-Year Cohort Study. Journal of Nutrition, Health and Aging. 2021;25(1):108-15.

107. Zhang S, Gu Y, Rayamajhi S, Thapa A, Meng G, Zhang Q, et al. Ultra-processed food intake is associated with grip strength decline in middle-aged and older adults: a prospective analysis of the TCLSIH study. European Journal of Nutrition. 2022;61(3):1331-41.

108. Perri G, Mendonça N, Jagger C, Walsh J, Eastell R, Mathers JC, et al. Dietary Selenium Intakes and Musculoskeletal Function in Very Old Adults: Analysis of the Newcastle 85+ Study. Nutrients. 2020;12(7).

109. Sahni S, Dufour AB, Fielding RA, Newman AB, Kiel DP, Hannan MT, et al. Total carotenoid intake is associated with reduced loss of grip strength and gait speed over time in adults: The Framingham Offspring Study. The American journal of clinical nutrition. 2021;113(2):437-45.

110. Granic A, Jagger C, Davies K, Adamson A, Kirkwood T, Hill TR, et al. Effect of Dietary Patterns on Muscle Strength and Physical Performance in the Very Old: Findings from the Newcastle 85+ Study. PloS one. 2016;11(3):e0149699.

111. Yokoyama Y, Nishi, M., Murayama, H., Amano, H., Taniguchi, Y., Nofuji, Y., Narita, M., Matsuo, E., Seino, S., Kawano, Y., Shinkai, S. Dietary variety and decline in lean mass and physical performance in community-dwelling older Japanese: A 4-year follow-up study. The Journal of Nutrition, Health & Aging. 2017;21:11-6.

112. Wang TY, Wu Y, Wang T, Li Y, Zhang D. A prospective study on the association of sleep duration with grip strength among middle-aged and older Chinese. Experimental gerontology. 2018;103:88-93.

113. Cooper R, Mishra G, Clennell S, Guralnik J, Kuh D. Menopausal status and physical performance in midlife: findings from a British birth cohort study. Menopause (New York, NY). 2008;15(6):1079-85.

114. Shin HE, Walston JD, Kim M, Won CW. Sex-Specific Differences in the Effect of Free Testosterone on Sarcopenia Components in Older Adults. Frontiers in endocrinology. 2021;12:695614.

115. Paster IC, Lin P-ID, Rifas-Shiman SL, Perng W, Chavarro JE, Oken E. Association of total lifetime breastfeeding duration with midlife handgrip strength: findings from Project Viva. BMC women's health. 2022;22(1):306.

116. de Keijzer C, Tonne C, Sabia S, Basagaña X, Valentín A, Singh-Manoux A, et al. Green and blue spaces and physical functioning in older adults: Longitudinal analyses of the Whitehall II study. Environment international. 2019;122:346-56.

117. Charles LE, Burchfiel CM, Fekedulegn D, Kashon ML, Ross GW, Sanderson WT, et al. Occupational and other risk factors for hand-grip strength: the Honolulu-Asia Aging Study. Occupational and environmental medicine. 2006;63(12):820-7.
